# FBXO7, a tumor suppressor in endometrial carcinoma, suppresses INF2-associated mitochondrial division

**DOI:** 10.1038/s41419-023-05891-0

**Published:** 2023-06-21

**Authors:** Hui Zhang, Yiting Zhao, Jie Wang, Jinyun Li, Jingyi Xia, Yan Lin, Yeling Zhong, Xinyi Cao, Jiabei Jin, Xinming Li, Weili Yang, Meng Ye, Xiaofeng Jin

**Affiliations:** 1 Department of Biochemistry and Molecular Biology, Zhejiang Key Laboratory of Pathophysiology, Health Science Center, Ningbo University, Ningbo, Zhejiang 315211 China; 2 Department of Oncology, The First Hospital of Ningbo University, Ningbo University, Ningbo, Zhejiang 315020 China; 3 Department of Gynecology, The Affiliated People’s Hospital of Ningbo University, Ningbo, 315040 China

**Keywords:** Cancer genetics, Oncogenes

## Abstract

Endometrial carcinoma (ECa) is the most common malignant gynecological cancer, with an increased incidence and fatality rate worldwide, while the pathogenesis is still largely unknown. In this study, we confirmed that *FBXO7*, a gene coding FBXO7 E3 ubiquitin ligase, is significantly downregulated and mutated (5.87%; 31/528) in ECa specimens, and the abnormal low expression and mutations of *FBXO7* are associated with the occurrence of ECa. We also identify the excessive expression of INF2 protein, a key factor that triggers mitochondrial division by recruiting the DRP1 protein, and the elevated INF2 protein is significantly negatively correlated with the low FBXO7 protein in ECa specimens. Mechanistically, FBXO7 restrains ECa through inhibiting INF2-associated mitochondrial division via FBXO7-mediated ubiquitination and degradation of INF2. Moreover, we found that ECa-associated FBXO7 mutants are defective in the ubiquitination and degradation of INF2, promoting ECa cells proliferation, migration and apoptosis inhibition via inducing mitochondrial hyper-division. In addition, we found that it could reverse *FBXO7* deletion or ECa-associated FBXO7 mutants-induced proliferation, migration, apoptosis inhibition and mitochondrial hyper-division of ECa cells by *INF2* or *DNM1L* knockdown, or DRP1 inhibitor Mdivi-1. In summary, our study shows that FBXO7 acts as a novel tumor suppressor in ECa by inhibiting INF2-DRP1 axis-associated mitochondrial division through the ubiquitination and degradation of INF2 while the effect is destroyed by ECa-associated FBXO7 and INF2 mutants, highlights the key role of FBXO7-INF2-DRP1 axis in ECa tumorigenesis and provides a new viewpoint to treat ECa patients with *FBXO7* deletion or mutations by targeting INF2-DRP1 axis-associated mitochondrial division.

## Introduction

Endometrial carcinoma (ECa) is the most common gynecological malignant cancer, with an increased incidence and fatality rate in developed countries [1]. Some patients with ECa can be completely cured with surgery, but the outcome of patients with advanced ECa is always poor, requiring a more efficient clinical treatment strategy [2]. The diagnosis of ECa relies on histopathology, which could not identify the tumor heterogeneity of ECa or improve the efficacy of the ECa treatment [3]. Diagnosis of ECa using molecular determinants, including p53, endometrial receptor α (ERα), progesterone receptor (PR), and others, have exhibited an improved efficacy of ECa treatment [4]. Ongoing studies on novel molecular targets that are unique and sensitive may further improve the outcome of ECa treatment.

Disruption of the ubiquitin-proteasome system (UPS) results in inadequate degradation of oncoproteins, leading to the occurrence and progression of cancers [5]. The UPS consists of a three-step enzymatic reaction mediated by ubiquitin-activating enzyme (E1), ubiquitin-conjugating enzyme (E2), and E3 ubiquitin ligase, culminating in the degradation of the protein through the proteasome [6]. E3 ubiquitin ligases are the most abundant, especially the CULLIN (CUL) protein family (CUL1, CUL2, CUL3, CUL4A, CUL4B, CUL5, CUL7, and CUL9), which specifically recognizes substrates and plays an important role in the regulation of protein homeostasis [7], and E3 ubiquitin ligases mutations are always involved in the occurrence and progression of cancers [8].

The CUL1 E3 ubiquitin ligase complex consists of the scaffold protein CUL1, an adaptor protein generally F-box proteins (FBP), S-phase kinase-associated protein 1 (SKP1), which mediates the binding of adaptor protein to CUL1, and RING box protein 1 (Rbx1), which mediates the binding of E2 to CUL1 [9]. F-box-only protein 7 (FBXO7) is an adaptor protein of the CUL1 E3 ubiquitin ligase complex that functions as a substrate recognition subunit [10]. Previous studies have shown that FBXO7 acts as a promoter of mitophagy to remove damaged mitochondria, thus preventing Parkinson’s disease (PD), while PD-associated FBXO7 mutants promote the occurrence of PD [11, 12]. Notably, *FBXO7* was highly mutated in ECa (5.87%; 31/528; data from the Cancer Genome Atlas (TCGA); https://portal.gdc.cancer.gov/). However, the functions of FBXO7 and the effects of ECa-associated FBXO7 mutants on ECa remain unknown.

The deregulation of cellular energetics, a typical feature of cancer, is mainly caused by mitochondrial dysfunction [13]. Mitochondria are highly dynamic organelles that maintain a balance between division and fusion [14]. Unbalanced mitochondrial division and fusion, especially division, is closely associated with cancer tumorigenesis and may induce malignant cancers [15]. Classic mitochondrial division is mediated by the accumulation of dynamin-related protein 1 (DRP1) in an endoplasmic reticulum (ER)-dependent manner [16]. Mechanistically, ER-localized inverted formin 2 (INF2) induces actin polymerization and recruits DRP1 in mitochondrial-ER contacts, triggering midzone division of mitochondria [16, 17]. Mitochondrial hyper-division caused by INF2 in prostate cancer (PCa) has been shown to be involved in the migration and invasion of PCa cells [18, 19]. Besides, the phosphorylation modification of DRP1 also could regulate its activity of mitochondrial division. For example, DRP1 Ser616 phosphorylation could dramatically contribute to mitochondrial division, while the Ser637 phosphorylation confers the inverse effect [20]. However, the phosphorylation of DRP1 and the role of abnormal expression of INF2-induced unbalanced mitochondrial division and fusion in the occurrence and progression of ECa still remain elusive.

Here, we demonstrate that FBXO7 induces the ubiquitination and degradation of INF2, while the effect is destroyed by ECa-associated FBXO7 and INF2 mutants. Moreover, we find that the *FBXO7* deletion and mutations lead to the accumulation of INF2 thus inducing the DRP1 assembling or/and Ser616 phosphorylation to contribute to the mitochondria hyper-division, and results in the excessive proliferation, migration, and suppressive apoptosis of ECa cells. Finally, we corroborated that *FBXO7* deletion or ECa-associated mutants induced proliferation, migration, apoptosis inhibition and mitochondrial hyper-division of cells by regulating the INF2-DRP1 axis including the deletion of *INF2* and *DNM1L* (gene name of DRP1), and applying the DRP1 inhibitor Mdivi-1. Our findings support that FBXO7 negatively regulates INF2-DRP1 axis to inhibit mitochondria division and acts as a tumor suppressor in ECa.

## Results

### Identification of FBXO7 as a tumor suppressor in ECa

**Fig. 1:**
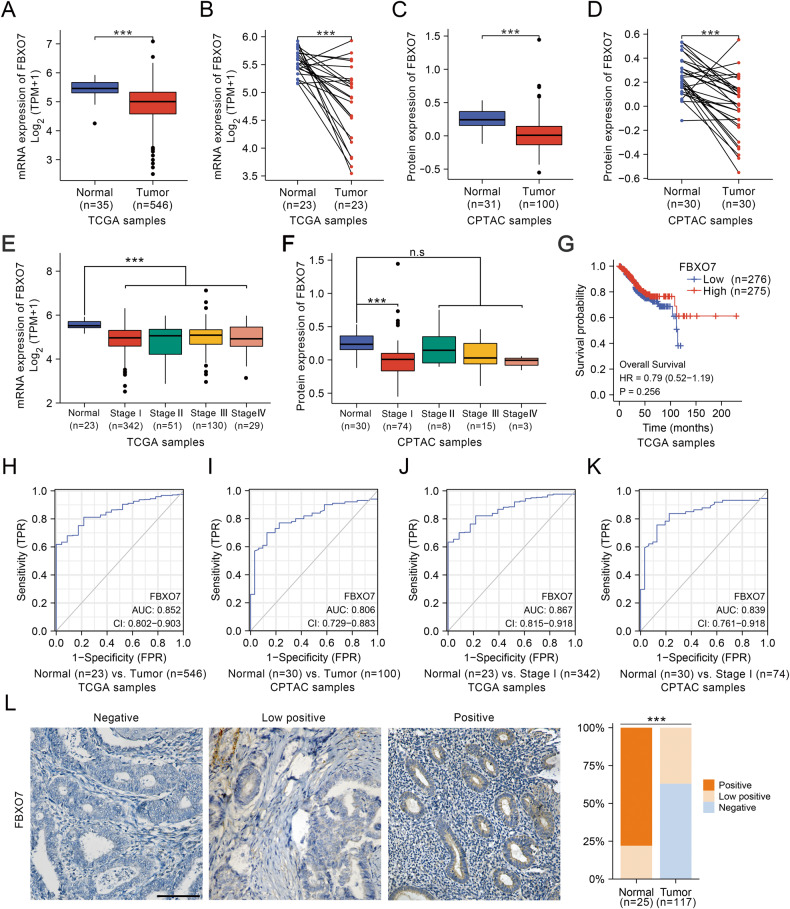
*FBXO7* mRNA and FBXO7 protein expression level are downregulated in ECa patients. **A** *FBXO7* mRNA expression level in ECa samples (*n* = 546) and normal endometrium samples (*n* = 35) from TCGA database using R software. ***p < 0.001 vs. the Normal group. **B** *FBXO7* mRNA expression level in the ECa samples (*n* = 23) and corresponding normal endometrium samples from TCGA database using R software. ***p < 0.001 vs. the Normal group. **C** FBXO7 protein expression level in ECa samples (*n* = 100) and normal endometrium samples (*n* = 31) from CPTAC database using R software. ***p < 0.001 vs. the Normal group. **D** FBXO7 protein expression level in the ECa samples (*n* = 30) and corresponding normal endometrium samples from CPTAC database using R software. ***p < 0.001 vs. the Normal group. **E** Correlation between *FBXO7* mRNA expression level and clinical stage of ECa (Normal = 23, Stage I = 342, Stage II = 51, Stage III = 130, Stage IV = 29) from TCGA database using R software. ***p < 0.001 vs. the Normal group. **F** Correlation between FBXO7 protein expression level and clinical stage of ECa (Normal = 30, Stage I = 74, Stage II = 8, Stage III = 15, Stage IV = 3) from CPTAC database using R software. ***p < 0.001 vs. the Normal group. **G** Correlation between *FBXO7* mRNA expression level (High = 275, Low =276) and prognosis in ECa patients from TCGA database using R software. High- and low-expression groups were defined by the median *FBXO7* mRNA expression level of the study population. **H** ROC analysis of *FBXO7* mRNA to normal (*n* = 23) and tumor (*n* = 546) subgroup of ECa from TCGA database using R software. **I** ROC analysis of FBXO7 protein to normal (*n* = 30) and tumor (*n* = 100) subgroup of ECa from CPTAC database using R software. **J** ROC analysis of *FBXO7* mRNA to normal (*n* = 23) and stage I (*n* = 342) subgroup of ECa from TCGA database using R software. **K** ROC analysis of FBXO7 protein to normal (*n* = 30) and stage I (*n* = 74) subgroup of ECa from CPTAC database using R software. **L** Representative images (Left) and staining patterns (Right) of IHC analysis of FBXO7 protein expression on ECa and normal endometrium tissues (142 samples, including 25 normal tissues and 117 ECa tissues). Scale bar, 100 μm. ***p < 0.001 vs. the Normal group.

The ECa database from The Cancer Genome Atlas (TCGA) shows that the low expression of *FBXO7* mRNA in ECa samples compared to normal samples (Fig. 1A, B). Similar results of FBXO7 protein expression level are found in the ECa database from Clinical Proteomic Tumor Analysis Consortium (CPTAC) (Fig. 1C, D), suggesting that FBXO7 may act as a tumor suppressor in ECa. Further analysis from these two databases shows that *FBXO7* mRNA level and FBXO7 protein level are not correlated with the clinical stage and prognosis of ECa (Fig. 1E–G). And the receiver operating characteristic curve (ROC) analyses show the area under curve (AUC) value of 0.852 for *FBXO7* mRNA and 0.806 for FBXO7 protein to normal and tumor subgroup, respectively (Fig. 1H, I), suggesting that FBXO7 is a potential marker for ECa diagnosis. Notably, the AUC values of either mRNA or protein level of FBXO7 between normal and stage I subgroup are higher, supporting that FBXO7 may be involved in the occurrence of ECa (Fig. 1J, K). We also analyzed the expression of FBXO7 protein via immunohistochemistry (IHC) in our collection of human endometrial normal tissue (25 samples) and cancer tissue samples (117 samples). And results are consistent with the data from CPTAC that FBXO7 protein is significantly expressed at a lower level in cancer tissues (Fig. 1L). These data suggest that FBXO7 is a tumor suppressor and may be involved in the occurrence of ECa.

### Identification of INF2 as a novel FBXO7 interacting protein

**Fig. 2:**
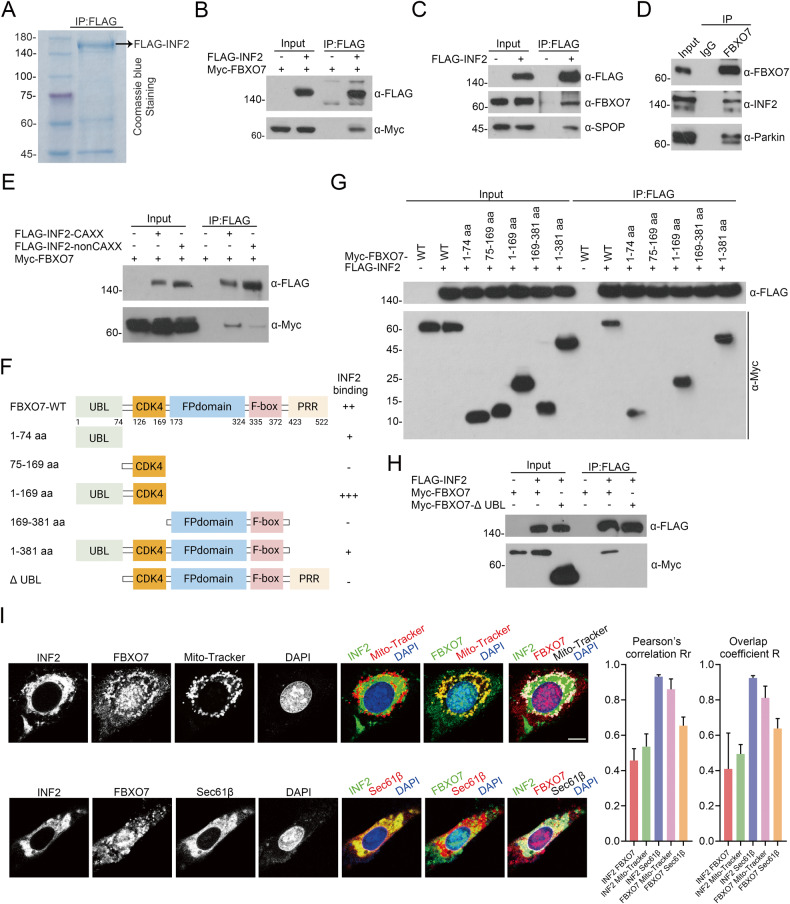
Identification of INF2-FBXO7 protein complex. **A** FLAG-INF2 protein complex are obtained from HEK-293T cells through co-IP of anti-FLAG antibody and detected by Coomassie Blue staining. **B** Western blotting of whole cell lysates (WCLs) and co-IP samples of anti-FLAG antibody obtained from HEK-293T cells transfected with Myc-FBXO7 plasmid and/or not FLAG-INF2 plasmid, and treated with 20 μM MG132 for 8 h before harvesting. **C** Western blotting of WCLs and co-IP samples of anti-FLAG antibody obtained from HEK-293T cells transfected with FLAG-INF2 plasmid or not. SPOP is a known interacting protein of INF2. **D** Western blotting of WCLs and co-IP samples of anti-FBXO7 antibody obtained from AN3 CA cells. Parkin as a known interacting protein of FBXO7. **E** Western blotting of WCLs and co-IP samples of anti-FLAG antibody obtained from HEK-293T cells transfected with Myc-FBXO7 plasmid and/or not FLAG-INF2-CAXX/nonCAXX plasmids treated with 20 μM MG132 for 8 h before harvesting. **F** Diagram showing FBXO7-WT and FBXO7 structural domain truncation mutants. **G** Western blotting of WCLs and co-IP samples of anti-FLAG antibody obtained from HEK-293T cells transfected with Myc-FBXO7-WT/1-74 aa/75-169 aa/1-169 aa/169-381 aa/1-381 aa plasmids and/or not FLAG-INF2 plasmid, and treated with 20 μM MG132 for 8 h before harvesting. **H** Western blotting of WCLs and co-IP samples of anti-FLAG antibody obtained from HEK-293T cells transfected with Myc-FBXO7-WT/ΔUBL plasmids and/or not FLAG-INF2 plasmid, and treated with 20 μM MG132 for 8 h before harvesting. **I** Representative immunofluorescence images (Left) of U-2OS cells transfected with pCMV-mCherry-Sec61β plasmid or treated with Mito-Tracker Red, stained with FBXO7, INF2, Sec61β or Mito-Tracker, and DAPI. Quantitative analysis (Right) of colocalization among INF2, FBXO7, Sec61β and Mito-Tracker through Image Pro Plus 6.0 software. Pearson’s correlation coefficient is the most common correlation coefficient to quantitatively describe colocalization, the value is between 1 and –1. 1 is a perfect correlation (where there is protein A, there must be protein B); –1 means complete exclusion (where there is protein A, there must be no protein B), and 0 means a random relationship (protein A and protein B are distributed randomly). Scale bar, 20 μm. Experiments in (**B**, **C**, **D**, **E**, **G**, **H**, **I**) were repeated three times.

The affinity-purification mass spectrometry (AP-MS) result from two proteomics studies hint the potential interacted proteins of FBXO7 including INF2, a key factor in DRP1-associated mitochondrial division [21, 22]. A similar result is presented in the AP-MS data of our group via analyzing the INF2 protein complex, which is obtained by stably expressing FLAG-INF2 in HEK-293T cells, verifying the potential interaction between FBXO7 and INF2 (Table 1 and Fig. 2A). Previous studies have revealed that FBXO7 regulates the quality of mitochondria via the promotion of mitophagy [10], while limited studies have revealed the relationship between FBXO7 and mitochondrial division. To authenticate that INF2 is a bona fide FBXO7 interacting protein, we investigated the interaction using a co-immunoprecipitation (co-IP) assay. Co-IP assay shows that Myc-FBXO7 interacted with FLAG-INF2 (Fig. 2B), and FLAG-INF2 can capture endogenous FBXO7, as well as a known interacting protein, SPOP, in HEK-293T cells (Fig. 2C). In addition, we obtained the endogenous FBXO7-INF2 protein complex and the well-known FBXO7-Parkin protein complex in AN3 CA cells through co-IP (Fig. 2D). In addition, in order to test whether INF2 is a specific substrate for FBXO7 or not, we tested the interaction of INF2 with several other FBXO family members, including FBXO11, FBXO31, F-box and WD repeat domain-containing 7 (FBXW7). Co-IP assay shows that only Myc-FBXO7 interacts with FLAG-INF2 but not others (Supplementary Fig. 1). Besides, we also tried to mimic the binding ability of INF2 to FBXO family members, and we found FBXO4 (PDB ID: 3L2O), FBXO20 (PDB ID: 2EAQ) and FBXO44 (PDB ID: 3WSO) are in Protein Bank Data (PDB, https://www.rcsb.org/). Then we use the Z-DOCK software to analyze the affinity of FBXO7 (PDB ID: 4L9H) and those FBXO family members to INF2, respectively, and Table 2 shows that the highest affinity of FBXO7 with INF2 among these FBXO family members. In addition, digging deep into our data of AP-MS, some FBX family members, including FBXO3, FBXO7, FBXO50, F-box/LRR-repeat protein 18 (FBXL18), and FBXL20 were captured by FLAG-INF2. The affinity of these FBX family members to INF2 proteins was ranked according to the number of unique peptides detected. We can see that FBXO7 ranks first among FBXO subfamily members, suggesting a strong interaction between FBXO7 and INF2 (Table 3).

**Table 1:** The number of total/unique peptides identified by mass spectrometry analysis.

Group	Protein name	FLAG-INF2	
		Peptide count	Unique peptide count
Novel	FBXO7	3	3
	PDK1	3	3
	MCM3	10	10
Known	Actin	31	1
	CAP1	3	3
	ADSS	4	4

**Table 2:** The affinity analysis between FBXO family members and INF2 through Z-DOCK.

Rank	Protein name	PDB ID	Z-score	Z-rank score
1	FBXO7	4L9H	20.46	–80.391
2	FBXO20	2EAQ	13.4	–71.393
3	FBXO44	3WSO	26.42	–6.641
4	FBXO4	3L2O	21.64	–6.563

**Table 3:** The rank of FBX family members in AP-MS through unique peptides.

Rank	Protein name	FLAG-INF2	
		Peptide count	Unique peptide count
1	FBXO7	3	3
2	FBXO3	1	1
	FBXO50	1	1
	FBXL18	1	1
	FBXL20	1	1

In eukaryotic cells, INF2 has two C-terminal splice variants: ER-localized INF2, also known as INF2-CAXX, and INF2-nonCAXX [19]. We found that Myc-FBXO7 co-immunoprecipitated with both INF2-CAXX and INF2-nonCAXX (Fig. 2E). Considering that both INF2-CAXX and FBXO7 substantially alter mitochondrial function, we focused on the relationship between INF2-CAXX (hereafter referred to as INF2) and FBXO7 in the following study. Then, we generated a series of FBXO7 truncation mutants to explore the key domain which is recognized by INF2 (Fig. 2F), and the co-IP assay shows that the FBXO7 mutants that retain the ubiquitin-like (UBL) domain (1–74 aa) still bound to INF2 (Fig. 2G), implying that the UBL domain is critical for the FBXO7-INF2 binding. Accordingly, we also generated an FBXO7 mutant with a deletion of the UBL domain, which was named as FBXO7-ΔUBL (Fig. 2F). As expected, in vivo interaction assays show that FBXO7-ΔUBL failed to capture INF2 (Fig. 2H), confirming that the UBL domain is the key domain of FBXO7 in binding to INF2.

Then, we investigated the subcellular localization of FBXO7-INF2 interaction. As shown in Fig. 2I, INF2 is located in the cytoplasm and closely binds to Sec61β (ER marker), but it not so perfectly colocalizes with Mito-Tracker (mitochondria marker) (Fig. 2I). Compared to INF2, we found that FBXO7 protein is prone to colocalize with mitochondria than ER. In addition, FBXO7 partly colocalized with INF2, and FBXO7-INF2 protein complex appears to be mainly located in the ER (Fig. 2I). In summary, we identified an interaction between INF2 and FBXO7.

### Identification of INF2 as a degradative substrate of FBXO7

**Fig. 3:**
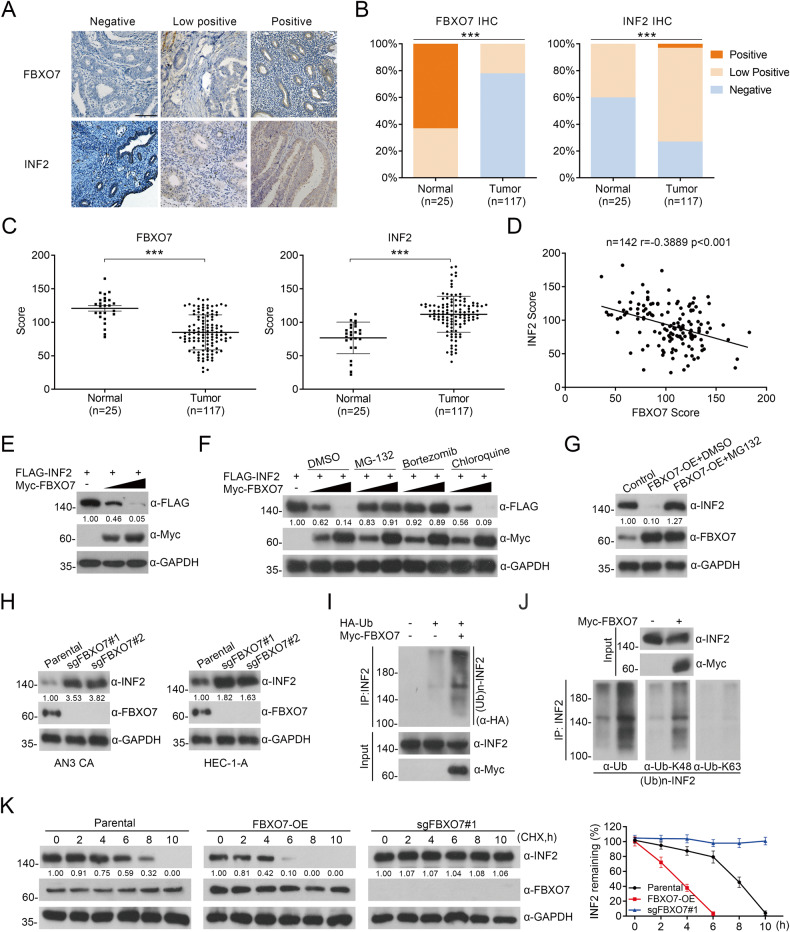
FBXO7 protein mediates the degradative ubiquitination of INF2 protein. **A** Representative images of IHC analysis of FBXO7 and INF2 proteins expression on ECa and normal endometrium tissues (142 samples, including 25 normal tissues and 117 ECa tissues). Scale bar, 100 μm. ***p < 0.001 vs. the Normal group. **B** Staining patterns of IHC analysis based on (**A**). ***p < 0.001 vs. the Normal group. **C** The score of FBXO7 (Left) and INF2 (Right) proteins expression levels based on (**A**). ***p < 0.001 vs. the Normal group. **D** Correlation analysis of the protein expression levels based on (**A** and **C**). *r* = –0.3889, *p* < 0.001. **E** Western blotting of WCLs of HEK-293T cells transfected with FLAG-INF2 plasmid and the increasing Myc-FBXO7 plasmid. All quantitation were normalized to the protein level of endogenous control GAPDH. **F** Western blotting of WCLs of HEK-293T cells transfected with transfected with FLAG-INF2 plasmid and the increasing Myc-FBXO7 plasmid, and treated with MG132 (20 μM), Bortezomib (200 nM), Chloroquine (100 mM) or DMSO for 8 h before harvesting. All quantitation were normalized to the protein level of endogenous control GAPDH. **G** Western blotting of WCLs of AN3 CA cells transfected with pCDH-CD513B-FBXO7 plasmid and treated with MG132 (20 μM) or DMSO for 8 h before harvesting. All quantitation were normalized to the protein level of endogenous control GAPDH. **H** *FBXO7* knockout was performed in AN3 CA and HEC-1-A cell lines. Western blotting of WCLs of AN3 CA (Left) and HEC-1-A (Right) cells with *FBXO7* knockout and parental. All quantitation were normalized to the protein level of endogenous parental GAPDH. **I** Western blotting of the products of in vivo ubiquitination assays performed using WCLs and co-IP samples of anti-INF2 antibody from AN3 CA cells transfected with HA-Ub and Myc-FBXO7 plasmids or not, and treated with 20 μM MG132 for 8 h before harvesting to show the ubiquitination of endogenous INF2 protein mediated by FBXO7 protein. **J** Western blotting of the products of in vivo ubiquitination assays performed using WCLs and co-IP samples of anti-INF2 antibody from HEK-293T cells transfected with Myc-FBXO7 plasmid or not, and treated with 20 μM MG132 for 8 h before harvesting to show the ubiquitin linkage specificity of endogenous INF2 protein mediated by FBXO7 protein. **K** The half-life of INF2 protein was detected by western blotting of WCLs of AN3 CA cells with FBXO7 overexpression, *FBXO7* knockout and parental treated with 50 μg/ml cycloheximide (CHX) and harvested at different time points. All quantitation were normalized to the protein level of endogenous parental GAPDH (Left). Statistics of INF2 protein half-life (Right). Data are shown as means ± SD (*n* = 5). Experiments in (**E**, **F**, **G**, **H**, **I**, **J**) were repeated three times.

FBXO7 is an adaptor protein of the CUL1 E3 ubiquitin ligase complex that promotes the ubiquitination and degradation of substrates [10]. Thus, we analyzed whether FBXO7 could promote ubiquitination and degradation of INF2. Firstly, we analyzed the expression of FBXO7 and INF2 proteins via IHC in human ECa specimens. We found that INF2 is significantly overexpressed in tumor tissues (Fig. 3A–C). It is worth noting that statistical analysis indicates that FBXO7 protein was negatively correlated with INF2 protein expression in these tissues (Fig. 3A–D; *r* = –0.3889, *p* < 0.001). Then, we show that exogenous FBXO7 decreases the protein level of exogenous INF2 in a dose-dependent manner (Fig. 3E). And both endogenous and exogenous INF2 degradation induced by FBXO7 are completely blocked when cells are treated with the proteasome inhibitors MG132 or Bortezomib (Fig. 3F, G). By contrast, the lysosome inhibitor Chloroquine has no effect on FBXO7-mediated INF2 degradation (Fig. 3F), indicating that FBXO7 down-regulates INF2 protein via the proteasomal- but not the lysosomal-degradation pathway. Correspondingly, we established *FBXO7* knockout AN3 CA and HEC-1-A cell lines (Fig. 3H). As Fig. 3H shows that *FBXO7* knockout results in the accumulation of INF2 protein in both AN3 CA cells and HEC-1-A cells. In addition, we selected one *FBXO7*-knockout cell line (sgFBXO7#1) from both AN3 CA cells and HEC-1-A cells to detect subsequent biological functions in tumor cell. In vivo ubiquitination assays reveal that endogenous INF2 is robustly polyubiquitinated by exogenous FBXO7 (Fig. 3I). Then, we examined the linkage specificity of FBXO7-mediated INF2 ubiquitination using commercially available linkage-specific K48/63-Ub antibodies. K48-linked polyubiquitin chains generally trigger the degradation of substrates via the 26s proteasome, whereas K63-linked polyubiquitin chains are associated with signal transduction via non-degradative ubiquitination [7]. Corresponding to our previous results, in vivo ubiquitination assays indicate that K48-linked polyubiquitin chains were the main type of FBXO7-mediated INF2 ubiquitination (Fig. 3J). Moreover, we also found that the half-life of endogenous INF2 is significantly reduced by FBXO7 (Fig. 3K). Subsequently, the half-life of endogenous INF2 in AN3 CA cells are rescued by *FBXO7* knockout (Fig. 3K). All the results show that FBXO7-mediated polyubiquitination and degradation of INF2.

### ECa-associated FBXO7 and INF2 mutants are defective in FBXO7-INF2 interaction and FBXO7-mediated INF2 ubiquitination and degradation

**Fig. 4:**
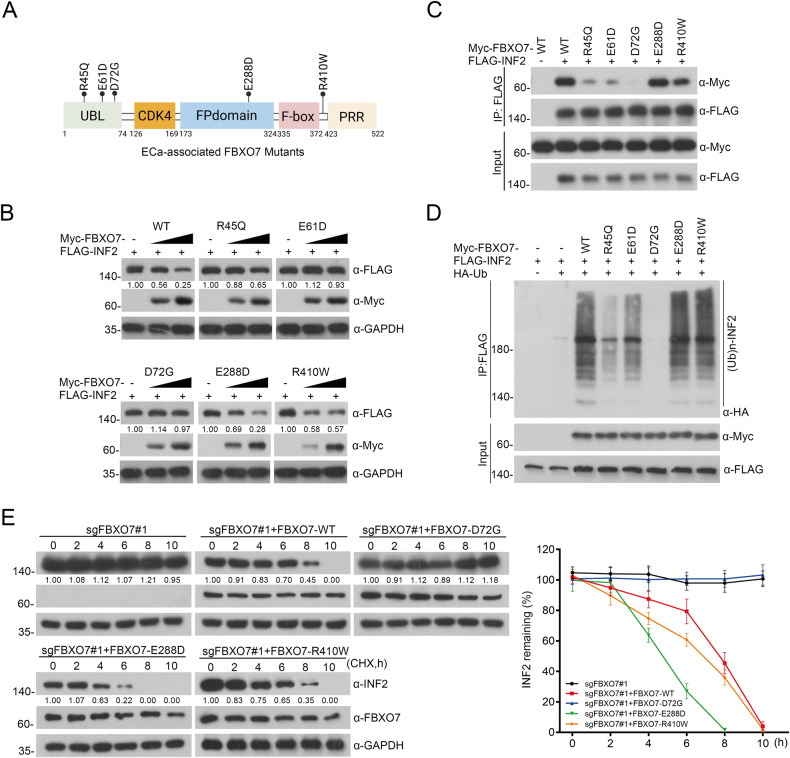
The accumulation of INF2 induced by ECa-associated FBXO7 mutants. **A** Diagram showing ECa-associated FBXO7 mutants from cBioportal database. **B** Western blotting of WCLs of HEK-293T cells transfected with FLAG-INF2 plasmid and the increasing Myc-FBXO7-WT/R45Q/E61D/D72G/E288D/R410W plasmids. All quantitation were normalized to the protein level of endogenous control GAPDH. **C** Western blotting of WCLs and co-IP samples of anti-FLAG antibody obtained from HEK-293T cells transfected with Myc-FBXO7-WT/R45Q/E61D/D72G/E288D/R410W plasmids and/or not Myc-FBXO7 plasmid, and treated with 20 μM MG132 for 8 h before harvesting. **D** Western blotting of the products of in vivo ubiquitination assays performed using WCLs and co-IP samples of anti-FLAG antibody from HEK-293T cells transfected with FLAG-INF2 plasmid, and/or not transfected with Myc-FBXO7-WT/R45Q/E61D/D72G/E288D/R410W and HA-Ub plasmids, and treated with 20 μM MG132 for 8 h before harvesting. **E** The half-life of INF2 protein was detected by western blotting of WCLs of AN3 CA cells with *FBXO7* knockout and transfected with Myc-FBXO7-WT/D72G/E288D/R410W plasmids, and treated with 50 μg/ml cycloheximide (CHX) and harvested at different time points. All quantitation were normalized to the protein level of endogenous parental GAPDH (Left). Statistics of INF2 protein half-life (Right). Data are shown as means ± SD (*n* = 5). Experiments in (**B**, **C**, **D**) were repeated three times.

The mutations of tumor suppressor gene often act as dominant-negative or loss-of-function mutations, thus leading to the occurrence of cancers. We mined data from TCGA, including 528 samples from ECa patients, with 31 samples with *FBXO7* mutations (5.87%; 31/528) (Supplementary Fig. 2A). Further analysis of the data shows that *FBXO7* mutations are not associated with prognosis or clinical stage of ECa, but the age of onset was significantly reduced in patients with the *FBXO7* mutations (Supplementary Fig. 2B–D), suggesting that *FBXO7* mutations may be similar to the low expression of *FBXO7* mRNA and FBXO7 protein in ECa, which is involved in ECa occurrence. Considering that the UBL domain significantly affects FBXO7-INF2 interaction (Fig. 2H), and FBXO7-ΔUBL is deficient in the ubiquitination and degradation of INF2 (Supplementary Fig. 3), we focused on three distinct FBXO7 mutants (R45Q, E61D and D72G) that are in the UBL domain (Fig. 4A), and we suspected that these ECa-associated mutants may lead to ECa through the destruction of FBXO7-INF2 interaction. In addition, we are also concerned about two other mutations, E288D and R410W. E288D is in the FP domain of FBXO7, which mediates the formation of a dimer or trimer of FBXO7; R410W is not in any of the domains of the FBXO7 protein. Thus, we also generated these two FBXO7 mutants (E288D and R410W) to compare whether the mutations in the UBL domain (R45Q, E61D and D72G) differ in FBXO7-INF2 interaction (Fig. 4A). Indeed, FBXO7-R45Q, E61D, and D72G, appear to entirely fail to degrade and bind to INF2 compared with the FBXO7-WT, whereas FBXO7-E288D and -R410W show retained degradation and binding (Fig. 4B, C). In vivo ubiquitination assay also reveals that FBXO7-mediated ubiquitination of INF2 is markedly attenuated by the R45Q, E61D, especially D72G, while E288D and R410W mutants still mediate the ubiquitination of INF2 (Fig. 4D). Besides, we also checked the change of the half-life of INF2 induced by FBXO7-D72G, E288D, and R410W. Consistently, compared with the FBXO7-WT, R410W and E288D, the half-life of INF2 is significantly prolonged by the FBXO7-D72G (Fig. 4E). In summary, these data demonstrate that ECa-associated FBXO7 mutants in the UBL domain abrogate INF2 degradation and led to it stabilization in ECa. And considering the complete function loss of FBXO7-D72G mediated interaction and ubiquitination of INF2, we chose it as the typical example of ECa-associated FBXO7 mutants, and it was subcloned into pCDH-CD513B-vetor for the subsequent study in ECa cells.

INF2 mutants are also generated to explore the binding domain recognized by FBXO7 (Supplementary Fig. 4A). In vivo interaction and ubiquitination assays showed that the INF2 mutants that retain 940–1249 aa are still recognized by FBXO7 (Supplementary Fig. 4B), while the INF2 mutant with the deletion of 940–1249 aa (INF2-1-940 aa) fails to be recognized, polyubiquitinated, and degraded by FBXO7 (Supplementary Fig. 4C–F), suggesting that FBXO7 may recognize certain motifs in the C-terminal domain of INF2. Thus, we searched for INF2 mutants in ECa and other cancers and noted three cancer-associated INF2 mutants with deletions or impact in 940–1249 aa of INF2, including two missense mutants (L1114I, two samples, one in ECa and the other in liver hepatocellular carcinoma; T1179M, two samples, one in ECa and the other in sarcoma) and one nonsense mutation (V530X, 13 samples, two in ECa, one in lung adenocarcinoma, seven in stomach adenocarcinoma, and three in colorectal adenocarcinoma) (Supplementary Fig. 4G). Intriguingly, cancer-associated INF2 mutants are stabilized because of impaired FBXO7-INF2 interaction and FBXO7-mediated INF2 polyubiquitination and degradation (Supplementary Fig. 4H–J). In conclusion, ECa-associated mutants of FBXO7 or INF2 have a pivotal impact on the FBXO7-INF2 interaction and FBXO7-mediated INF2 ubiquitination and degradation, leading to INF2 accumulation in ECa.

### FBXO7-WT suppresses ECa cells proliferation and migration rather than ECa-associated FBXO7 mutants partly in an INF2-DRP1 axis-dependent manner

**Fig. 5:**
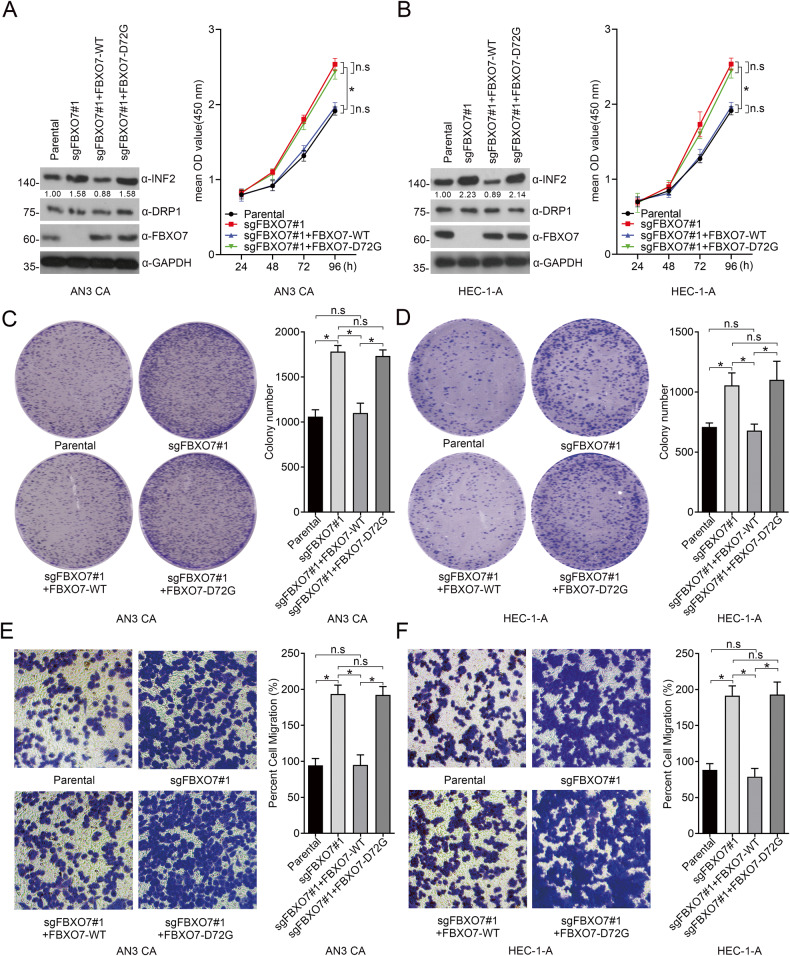
*FBXO7* knockout and FBXO7-D72G promote the proliferation and migration of ECa cells. **A** Western blotting of WCLs of AN3 CA cells with *FBXO7* knockout and transfected with pCDH-CD513B-FBXO7-WT/D72G plasmids, or parental. All quantitation were normalized to the protein level of endogenous parental GAPDH (Left). Cell proliferation assay of AN3 CA cells with *FBXO7* knockout and transfected with pCDH-CD513B-FBXO7-WT/D72G plasmids, or parental (Right). Data are shown as means ± SD (*n* = 5). *p < 0.05. **B** Western blotting of WCLs of HEC-1-A cells with *FBXO7* knockout and transfected with pCDH-CD513B-FBXO7-WT/D72G plasmids, or parental (Left). All quantitation were normalized to the protein level of endogenous parental GAPDH. Cell proliferation assay of AN3 CA cells with *FBXO7* knockout and transfected with FBXO7-WT/D72G, or parental (Right). Data are shown as means ± SD (*n* = 5). *p < 0.05. **C** Cell colony formation assay of AN3 CA cells with *FBXO7* knockout and transfected with pCDH-CD513B-FBXO7-WT/D72G plasmids, or parental (Left). Statistics of cell colony formation assay (Right). Data are shown as means ± SD (*n* = 3). *p < 0.05. **D** Cell colony formation assay of HEC-1-A cells with *FBXO7* knockout and transfected with pCDH-CD513B-FBXO7-WT/D72G plasmids, or parental (Left). Statistics of cell colony formation assay (Right). Data are shown as means ± SD (*n* = 3). *p < 0.05. **E** Cell migration assay of AN3 CA cells with *FBXO7* knockout and transfected with pCDH-CD513B-FBXO7-WT/D72G plasmids, or parental (Left). Statistics of cell migration assay (Right). Data are shown as means ± SD (*n* = 3). *p < 0.05. **F** Cell migration assay of HEC-1-A cells with *FBXO7* knockout and transfected with pCDH-CD513B-FBXO7-WT/D72G plasmids, or parental (Left). Statistics of cell migration assay (Right). Data are shown as means ± SD (*n* = 3). *p < 0.05. Experiments in (**A**, **B**) were repeated three times.

FBXO7 is reported to be a tumor suppressor and is involved in cell proliferation and migration in various cancer models [11]. Thus, we investigated whether FBXO7 also acts as a tumor suppressor in ECa. Indeed, *FBXO7* knockout dramatically triggers the proliferation and migration of AN3 CA and HEC-1-A cell lines (Fig. 5). And we observed that the stable overexpression of FBXO7-D72G mutant in these two cell lines fails to reverse the increased proliferation and migration of ECa cells, and the *FBXO7* knockout-induced INF2 accumulation (Fig. 5). These data suggest that *FBXO7* knockout and FBXO7-D72G may be involved in the occurrence and progression of ECa.

**Fig. 6:**
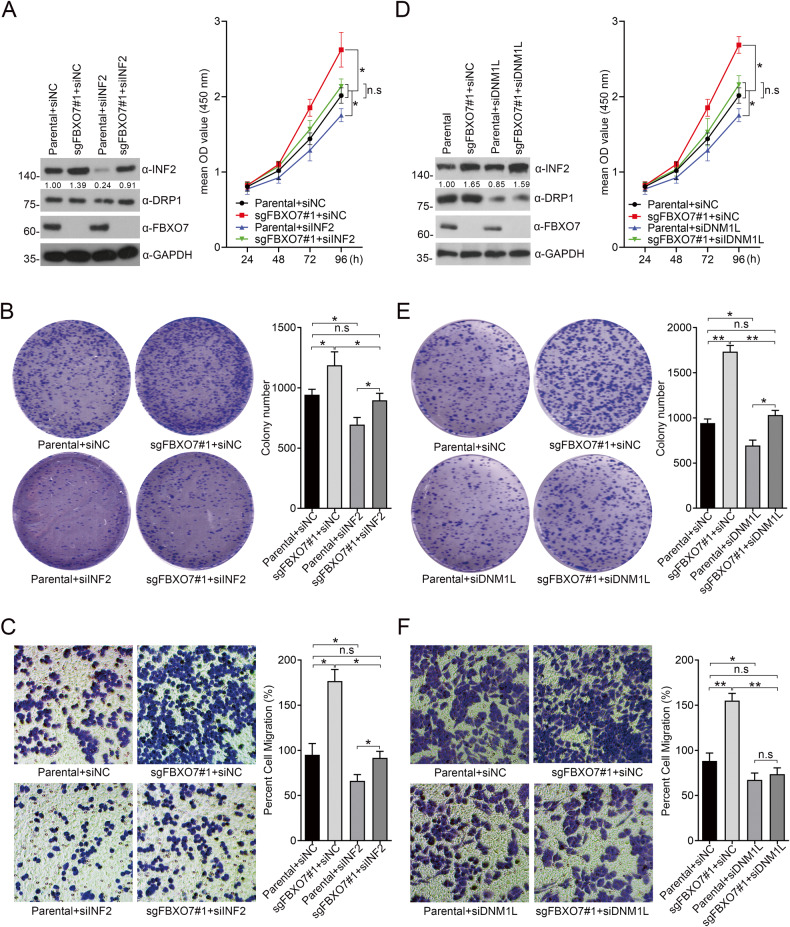
FBXO7 suppresses AN3 CA cells proliferation and migration partly in an INF2-DRP1 axis-dependent manner. **A** Western blotting of WCLs of AN3 CA cells with *FBXO7* knockout and transfected with siNC RNA, or si*INF2* RNA, or parental (Left). All quantitation were normalized to the protein level of endogenous parental GAPDH. Cell proliferation assay of AN3 CA cells with *FBXO7* knockout and transfected with siNC RNA, or si*INF2* RNA, or parental (Right). Data are shown as means ± SD (*n* = 5). *p < 0.05. **B** Cell colony formation assay of AN3 CA cells with *FBXO7* knockout and transfected with siNC RNA, or si*INF2* RNA, or parental (Left). Statistics of cell colony formation assay (Right). Data are shown as means ± SD (*n* = 3). *p < 0.05. **C** Cell migration assay of AN3 CA cells with *FBXO7* knockout and transfected with si*INF2* RNA, or si*INF2* RNA, or parental (Left). Statistics of cell migration assay (Right). Data are shown as means ± SD (*n* = 3). *p < 0.05. **D** Western blotting of WCLs of AN3 CA cells with *FBXO7* knockout and transfected with siNC RNA, or si*DNM1L* RNA, or parental (Left). All quantitation were normalized to the protein level of endogenous parental GAPDH. Cell proliferation assay of AN3 CA cells with FBXO7 knockout and transfected with siNC RNA, or si*DNM1L* RNA, or parental (Right). Data are shown as means ± SD (*n* = 5). *p < 0.05. **E** Cell colony formation assay of AN3 CA cells with *FBXO7* knockout and transfected with siNC RNA, or si*DNM1L* RNA, or parental (Left). Statistics of cell colony formation assay (Right). Data are shown as means ± SD (*n* = 3). *p < 0.05. **F** Cell migration assay of AN3 CA cells with *FBXO7* knockout and transfected with si*DNM1L* RNA, or si*DNM1L* RNA, or parental (Left). Statistics of cell migration assay (Right). Data are shown as means ± SD (*n* = 3). *p < 0.05, **p < 0.01. Experiments in (**A**, **D**) were repeated three times.

Considering the oncogenic functions of INF2 in cancers [19], we infer that FBXO7 may inhibit ECa cell proliferation and migration partly in an INF2-dependent manner. Indeed, the proliferation and migration of AN3 CA and HEC-1-A cells induced by *FBXO7* knockout was partly diminished by *INF2* knockdown (Fig. 6A–C and Supplementary Fig. 5A–C), indicating that *FBXO7* knockout promotes AN3 CA and HEC-1-A cells proliferation and migration, which is partly dependent on the accumulation of INF2 (Fig. 6A and Supplementary Fig. 5A). Previous study reported that INF2 induces actin polymerization and recruits DRP1, triggering pro-tumorigenic signals to induce proliferation and migration of PCa cells [18]. Thus, we hypothesized that FBXO7 partly inhibits the malignant phenotype of ECa cells by suppressing the INF2-DRP1 axis. Similar to *INF2* knockdown, *DNM1L* knockdown also rescues *FXBO7* knockout induced the malignant phenotype of ECa cells (Fig. 6D–F and Supplementary Fig. 5D–F), without affecting the INF2 protein level. In summary, our data support the notion that FBXO7 suppresses ECa cell proliferation and migration in an INF2-DRP1 axis-dependent manner (Fig. 6 and Supplementary Fig. 5).

### FBXO7 suppresses INF2-DRP1 axis-associated mitochondrial division

INF2 triggers the mitochondria division by recruiting DRP1 [16], but without a major change in mitochondrial function, including the basal mitochondrial reactive oxygen species (ROS) production, oxygen consumption rate (OCR), and membrane potential [18]. Thus, we reason that FBXO7 may play a tumor-suppressive role in ECa by modulating the INF2-DRP1 axis-associated mitochondrial division. In addition to INF2 and DRP1, we sought to investigate the effects of FBXO7 on the regulation of several pivotal markers involved in mitochondrial division and fusion, such as mitochondrial fission protein 1 (Fis1), mitochondrial fusion protein 1 (MFN1) and MFN2. The mRNA levels of those markers are not affected by the *FBXO7* knockout or overexpressed FBXO7 (Supplementary Fig. 6A). Of note, the MFN1 rather than MFN2 protein level was decreased after *FBXO7* knockout in both AN3 CA and HEC-1-A cells, which may be due to FBXO7-mediated non-degradative ubiquitination of MFN1 [12], suggesting that FBXO7 partly affects the process of mitochondrial fusion (Supplementary Fig. 6B). The evidence further supports our notion that FBXO7 acts as an inhibitor of mitochondrial division. To test our hypothesis, we first used Mito-Tracker Red dye to observe mitochondria in AN3 CA and HEC-1-A cells; however, the mitochondrial morphology of AN3 CA and HEC-1-A cells was not so clear (Supplementary Fig. 6C). U-2OS cells, with distinct mitochondrial morphology, is a common model for detecting and analyzing mitochondrial length [16], thus in this study, we also performed *FBXO7* knockout in U-2OS cells and attempted to use this cell model to investigate the effect of FBXO7 on mitochondrial division (Supplementary Fig. 6D).

**Fig. 7:**
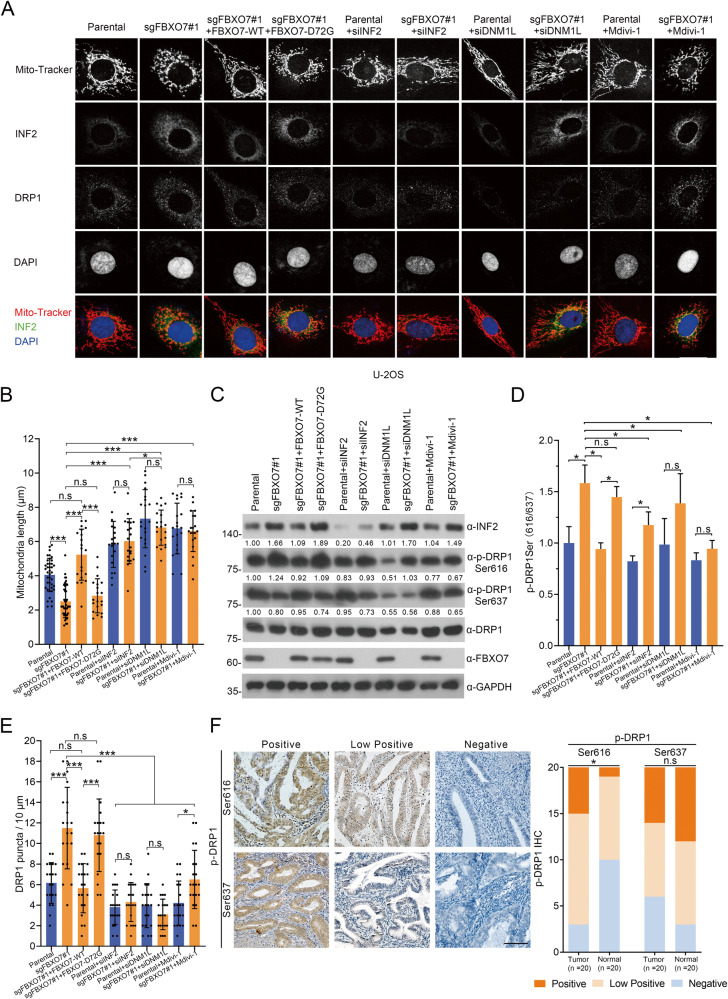
FBXO7 suppresses INF2-DRP1 axis-associated mitochondrial division. **A** Representative images of U-2OS cells with *FBXO7* knockout or parental transfected with pCDH-CD513B-FBXO7-WT/D72G plasmids, si*INF2* RNA, si*DNM1L* RNA, or treated with Mdivi-1 (20 μM). stained with INF2, DRP1, Mito-Tracker Red and DAPI. Scale bar, 20 μm. **B** Quantification of mitochondria lengths in (**A**). Data are shown as means ± SD (*n* = 20). *p < 0.05, ***p < 0.001. **C** Western blotting of WCLs of U-2OS cells in (**A**). The quantitation except DRP1 Ser616 and DRP1 Ser637 were normalized to the protein level of endogenous parental GAPDH. The quantitation of DRP1 Ser616 and DRP1 Ser637 phosphorylation were normalized to the protein level of endogenous parental DRP1. **D** Statistics of the ratio between DRP1 Ser616 to DRP1 Ser637. Data are shown as means ± SD (*n* = 3). *p < 0.05. **E** Quantification of DRP1 puncta in (**A**). Data are shown as means ± SD (*n* = 20). *p < 0.05, ***p < 0.001. **F** Representative images (Left) and staining patterns (Right) of IHC analysis of DRP1 Ser616 and DRP1 Ser637 expression on ECa (*n* = 20) and corresponding normal endometrium tissues. Scale bar, 100 μm. *p < 0.001 vs. the Normal group. Experiments in (**C**) were repeated three times.

We observed that *FBXO7* knockout results in decreased average mitochondrial length, while stable overexpression of FBXO7-WT rather than FBXO7-D72G rescues *FBXO7* knockout-induced decreases in average mitochondrial length (Fig. 7A, columns 1–4; Fig. 7B, columns 1–4). These data with those in Fig. 5 suggest that the *FBXO7* knockout and FBXO7-D72G may induce ECa cell proliferation and migration partly through the mitochondrial hyper-division. In addition, the fluorescence intensity and protein level of INF2 appear to be stronger in *FBXO7* knockout U-2OS cells compared to the parental, and similar results are observed in cells with stable overexpression of FBXO7-D72G mutant (Fig. 7A, columns 1–4; Fig. 7C, columns 1–4), corresponding to our results that FBXO7 induces the degradation of INF2 in ECa cell lines, which suggests the similar cell biological functions of FBXO7 in ECa cell lines and U-2OS cells. Notably, the protein level of DRP1 does not appear to be affected by the change in INF2 protein levels, which is consistent with the previous finding that INF2 is a recruiter of DRP1 [16] (Fig. 7C, columns 1–4). Previous studies showed that the activity of DRP1 could be regulated by phosphorylation. For example, DRP1 Ser616 phosphorylation contributes to trigger the mitochondrial division, while DRP1 Ser637 phosphorylation confers the inverse effect [23, 24]. Thus, we tried to investigate whether the FBXO7-INF2 axis affects the phosphorylation of DRP1. Notably, the DRP1 Ser616 phosphorylation is increased in the *FBXO7* knockout U-2OS cells, while DRP1 Ser637 phosphorylation decreased (Fig. 7C, columns 1 and 2), and the ratio of DRP1 Ser616 to DRP1 Ser637 further supports that the DRP1 is activated (Fig. 7D, columns 1 and 2). Similarly, the effect was reserved by the overexpression of FBXO7-WT rather than FBXO7-D72G (Fig. 7C, columns 3 and 4), suggesting that the *FBXO7* knockout and FBXO7-D72G may induce mitochondrial hyper-division partly through the regulation in DRP1 phosphorylation and activity (Fig. 7C, columns 1–4). It is also reported that DRP1 is localized to cytoplasm and to mitochondrially associated puncta [16], and we find that the DRP1 puncta (number of puncta per 10 μm of mitochondria) increased markedly in *FBXO7* knockout and FBXO7-D72G overexpressed U-2OS cells, where DRP1 puncta may be recruited by accumulated INF2 (Fig. 7E, columns 1–4). In summary, *FBXO7* knockout or FBXO7-D72G result in excessive mitochondrial division may be caused by insufficient degradation of INF2-induced abnormal recruitment of DRP1 or/and elevated DRP1 Ser616 phosphorylation. Notably, we also checked the phosphorylation of DRP1 Ser616 and Ser637 in ECa and normal endometrial tissues and found that the elevated phosphorylation of DRP1 Ser616 in ECa tissues compared to normal endometrial tissues, while DRP1 Ser637 did not confer the significant difference (Fig. 7F).

In addition, the decreased average mitochondrial length induced by *FBXO7* knockout is restored by *INF2* knockdown and *DNM1L* knockdown, further proving that FBXO7 suppresses INF2-DRP1 axis-associated mitochondrial division (Fig. 7B, columns 1 and 2 and 5–8). There is no significant difference in average mitochondrial length, as well as DRP1 puncta, between *FBXO7* knockout U-2OS cells with *INF2* knockdown and parental with *INF2* knockdown, suggesting that FBXO7 may regulate mitochondrial division through INF2 (Fig. 7C, columns 5 and 6; Fig. 7E, columns 5 and 6). However, the phosphorylation level of DRP1 including Ser616 and Ser637 seems to be different between *FBXO7* knockout U-2OS cells with *INF2* knockdown and parental with *INF2* knockdown (Fig. 7C, columns 5 and 6; Fig. 7D, columns 5 and 6). Notably, *DNM1L* knockdown leads to the most significant reduction of DRP1 puncta and appears to reverse mitochondrial hyper-division more effectively than *INF2* knockdown U-2OS cells with *FBXO7* knockout (Fig. 7B, columns 5–8; Fig. 7E, columns 5–8), demonstrating that FBXO7-mediated inhibition of mitochondrial division is mainly dependent on the regulation of the INF2-DRP1 axis. Considering that there is no specific inhibitor against INF2, we chose the DRP1 inhibitor Mdivi-1 to reverse the mitochondrial hyper-division induced by *FBXO7* knockout. Indeed, Mdivi-1 significantly reversed the reduction in mean mitochondrial length caused by *FBXO7* knockout, like the result of *DNM1L* knockdown (Fig. 7B, columns 7–10). Notably, the decrease of DRP1 puncta (Fig. 7E, columns 9 and 10), the inhibition of DRP1 Ser616 phosphorylation (Fig. 7C, columns 9 and 10), and the reduced ratio of DRP1 Ser616 to DRP1 Ser637 (Fig. 7D, columns 9 and 10) in *FBXO7* knockout U-2OS cells with Mdivi-1 treatment and parental with Mdivi-1 treatment proving that Mdivi-1 efficiently inhibit the activity rather than the protein level of DRP1 to suppress the mitochondrial hyper-division (Fig. 7B, columns 9 and 10). Together, these data suggest that *FBXO7* knockout induces the INF2-DRP1 axis-associated mitochondrial hyper-division, which can be reversed by the DRP1 inhibitor Mdivi-1.

**Fig. 8:**
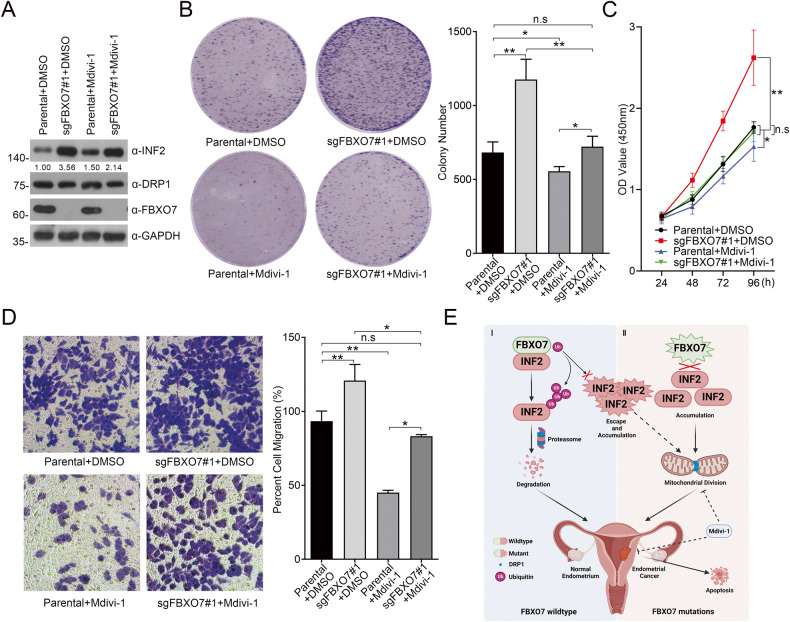
DRP1 inhibitor Mdivi-1 reverses the proliferation and migration of AN3 CA cells induced by *FBXO7* knockout. **A** Western blotting of WCLs of AN3 CA cells with *FBXO7* knockout or parental, and treated with DMSO or with Mdivi-1 (20 μM). All quantitation were normalized to the protein level of endogenous parental GAPDH. **B** Cell colony formation assay of AN3 CA cells with FBXO7 knockout or parental, and treated with DMSO or with Mdivi-1 (20 μM; Left). Statistics of cell colony formation assay (Right). Data are shown as means ± SD (*n* = 3). *p < 0.05, **p < 0.01. **C** Cell proliferation assay of AN3 CA cells with FBXO7 knockout or parental, and treated with DMSO or with Mdivi-1 (20 μM). Data are shown as means ± SD (*n* = 5). *p < 0.05. **D** Cell migration assay of AN3 CA cells with *FBXO7* knockout or parental, and treated with DMSO or with Mdivi-1 (20 μM; Left). Statistics of cell colony formation assay (Right). Data are shown as means ± SD (*n* = 3). *p < 0.05, **p < 0.01. **E** A model proposed according to the findings of the present study. I: Under physiological conditions, FBXO7-WT promptly degrades INF2 via UPS to prevent ECa. II: ECa-associated FBXO7 mutants are defective in the degradation of INF2 and ECa-associated INF2 mutants escape from the regulation by FBXO7, leading the INF2 accumulation and triggering the INF2-associated mitochondrial division, leading to ECa. Considering the FBXO7-INF2-DRP1 cascade, applying of Mdivi-1 may suppress proliferation and migration, and promote apoptosis of ECa cell lines with *FBXO7* deletion and mutations. Experiments in (**A**) were repeated three times.

Excessive mitochondrial division promotes the proliferation and migration of cancer cells; thus, previous studies have attempted to use Mdivi-1 to suppress the malignant phenotype of cancer cells [18, 25]. Indeed, Mdivi-1 could inhibit the *FBXO7* knockout-promoted proliferation and migration of AN3 CA cells (Fig. 8A–D). In summary, FBXO7 suppresses INF2-DRP1 axis-associated mitochondrial division, whereas abnormal low expression of FBXO7 or ECa-associated mutants would lead to the mitochondrial hyper-division, proliferation and migration of ECa cells, which could partly be able to be reversed by Mdivi-1.

## Discussion

In conclusion, we confirmed FBXO7 as a tumor suppressor in ECa by analyzing its expression data from ECa patients and detecting FBXO7-associated cell models. Mechanistically, we first authenticate FBXO7-mediated ubiquitination and degradation of INF2, while ECa-associated FBXO7 and INF2 mutants result in the defects of the formation of FBXO7-INF2 complex. Furthermore, our data indicate that the *FBXO7* deletion and ECa-associated mutants induced INF2 abundance, leading to INF2-DRP1 axis-induced mitochondrial hyper-division, and proliferation and migration of ECa cells. Notably, applying Mdivi-1 to ECa cell lines with abnormal low expression of FBXO7 or ECa-associated FBXO7 mutants could suppress ECa cells proliferation and migration (Fig. 8E). In addition, we show that ECa-associated INF2 mutants escape the regulation of FBXO7 and may lead to INF2 protein accumulation in ECa (Supplementary Fig. 4), while whether these INF2 mutants, especially INF2-V530X with the deletion at the C-terminal, still could promote the proliferation, migration and mitochondrial division of ECa cells is worthy to be studied. Additionally, Mdivi-1 also appears to inhibit the proliferation and migration of parental AN3 CA cells may partly be due to the Mdivi-1 in regulating reactive oxygen species in mitochondria [26]. However, considering that Mdivi-1 is the only specific DRP1 inhibitor, we have no other good choices to inhibit mitochondrial hyper-division induced by the high activity of INF2-DRP1 axis, suggesting that it is an urgent need to search for more efficient and specific molecules targeting this signal.

Previous studies reported that FBXO7 promotes mitophagy [10], which also be repeated in our study (Supplementary Fig. 7). We found that the colocalization between p62 puncta and mitochondria (Mito-Tracker) is increased through *FBXO7* knockout or with overexpression of FBXO7-D72G (Supplementary Fig. 7A, B), suggesting that the inhibition of mitophagy level by *FBXO7* knockout and FBXO7-D72G. Moreover, *DNM1L* knockdown and Mdivi-1 treatment show further inhibition of mitophagy, but not the *INF2* knockdown (Supplementary Fig. 7A, B).

A recent study identified two ways of mitochondrial division: midzone division and peripheral division [17]. Midzone division is a marker of cell proliferation, whereas peripheral division is enhanced under cell stress and it could trigger mitophagy [17]. Notably, mitochondrial division induced by the INF2-DRP1 axis is a typical common type of midzone division [16]. In this study, we identified that FBXO7 inhibits mitochondrial division by inducing ubiquitination and degradation of INF2, which is the first report to link midzone division to FBXO7. Superabundant fragmented and damaged mitochondria have been found in many cancer cells [27], representing excessive midzone division, insufficient peripheral division, and mitophagy. The effects of FBXO7 in inhibiting midzone division and promoting mitophagy provide evidence that FBXO7 functions as a tumor suppressor and is associated with mitochondrial dynamics.

In addition, mitochondrial division and DRP1 activity level are closely related to apoptosis level [28]. Inadequate apoptosis is a hallmark of cancer cells, and apoptosis level also has been used to evaluate the efficacy of targeted therapies [29]. Thus, we also evaluate the apoptosis of ECa cells after block of the FBXO7-INF2-DRP1 axis. Apoptosis is inhibited in *FBXO7* knockout HEC-1-A and AN3 CA cells (Supplementary Fig. 8A, B), which is dramatically reversed by FBXO7-WT, but not the FBXO7-D72G (Supplementary Fig. 8A, B). And *INF2* and *DNM1L* knockdown, as well as the treatment of Mdivi-1 all greatly reversed the apoptosis inhibition induced by *FBXO7* knockout. Moreover, Mdivi-1 treatment could promote the necrosis (extensive cytoplasmic membrane rupture and leakage of intracellular contents) and apoptosis (nuclear pyknosis with an integrated cytoplasmic membrane) of both HEC-1-A and AN3 CA cells compared to DMSO treatment (Supplementary Fig. 8C).

However, FBXO7 is still poorly studied in cancer biology and even in ubiquitination activity. Though increasing substrates of FBXO7 in cancer and nervous system diseases have been detected, such as Sirtuin 7 (SIRT7) [30], cyclin-dependent kinases (CDK6) [31] and Ubiquitously eXpressed Transcript isoform 2 (UXTV2) [32]. The substrate-binding consensus (SBC) motif is still not be identified in substrates of FBXO7. For example, the substrates of FBXW7 share the conserved CDC4 phosphodegron sequence 4-X-pThr (or pSer)-Pro-Pro-X-pSer (or pThr, Glu or Asp) (X represents any amino acid) [33]. Exploring whether similar consensus sequences (FBXO7 binding motifs) exist in substrates of FBXO7 will help to find more potential FBXO7 substrates in the future. In summary, our study shows the potential of FBXO7 in future cancer and mitochondrial studies.

## Materials and methods

### Cell lines and cell culture

The HEK-293T cell line (Cat No: TCH-C101) was obtained from Haixing Biosciences, Suzhou, Jiangsu, China. The endometrial carcinoma cell line (AN3 CA; Cat No: CL-0505) was obtained from Procell Life Science & Technology, Wuhan, Hubei, China. The endometrial carcinoma cell line (HEC-1-A) and human osteosarcoma cell line (U-2OS) were obtained from American Type Culture Collection (ATCC). HEK-293T was cultured in Dulbecco’s Modified Eagle Medium (DMEM, Meilunbio, China, Cat No: MA0212-2) with 10% Fetal Bovine Serum (FBS, Standard Quality, OriCell, China, Cat No: FBSST-01033). AN3 CA cell line was cultured in Minimum Essential Medium (MEM, Meilunbio, China, Cat No: MA0217) with 10% Fetal Bovine Serum (Standard Quality, OriCell, China, Cat No: FBSST-01033). U-2OS and HEC-1-A cell lines were cultured in McCoy’s 5A (Meilunbio, China, Cat No: MA0314) with 10% Fetal Bovine Serum (Standard Quality, OriCell, China, Cat No: FBSST-01033). All cells were grown at 37 °C with 5% CO_2_.

### Plasmid construction and transfections

The total primer sequences, including the construction and RT-qPCR primers, are listed in Supplementary Table 1.

The expression vectors for INF2-WT and mutants, and pCMV-mCherry-Sec61β have been described previously [18]. FBXO7 cDNA from HEK-293T cells was subcloned into the pCMV-Myc, pCMV-HA and pCDH-CD513B vector to construct pCMV-Myc-, pCMV-HA- and pCDH-CD513B-FBXO7 using specific primers. ECa-associated FBXO7 and INF2 mutants were generated using specific primers and overlap PCR. For knockout, sgRNA sequences targeting FBXO7 were subcloned into pCDH-CD513B-cas9-sgRNA vectors. sgRNA and siRNA sequences were listed in Supplementary Table 2. All constructs were verified by DNA sequencing.

All transfection experiments were performed using Lipo6000^TM^ transfection reagent (Beyotime, China, Cat No: C0526), according to the manufacturer’s instructions.

### Western blotting

Protein samples from each lysate from fresh cells treated with RIPA (Low) Lysis Buffer (Meilunbio, China, Cat No: MA0153) were loaded and separated by 10% SDS-PAGE and then transferred to Amersham Protran 0.2 μm nitrocellulose membranes (Cat No. 10600001; Cytiva, USA). NC membranes were blocked with 5% fat-free milk for 1 h at room temperature.

Membranes were probed with primary antibodies (Supplementary Table 3) at 4 °C overnight. The membranes were then incubated with HRP-conjugated secondary antibodies (Supplementary Table 3) for 1 h at room temperature. Proteins of interest were visualized using NcmECL Ultra (New Cell & Molecular Biotech, China, Cat No: P10300). WB was performed 2–3 times in at least two independent experiments, and representative pictures are shown.

### In vivo ubiquitination assay

HEK-293T cells were transfected with HA-ubiquitin or the indicated constructs. 36 h after transfection, the cells were treated with 20 μM MG132 for 8 h before harvesting. and then lysed in RIPA (Low) Lysis Buffer (Meilunbio, China, Cat No: MA0153). For immunoprecipitation, the cell lysates were incubated with anti-FLAG M2 agarose beads (Sigma, USA) or anti-INF2 Protein A/G immunoprecipitated magnetic beads (Bimake, China, Cat No: B23201) for 4 h at 4 °C. The bound beads were then washed four times with BC100 buffer (20 mM Tris-Cl, pH 7.9, 100 mM NaCl, 0.2 mM EDTA, 20% glycerol) containing 0.2% Triton X-100. The protein was eluted with FLAG peptide for 4 h at 4 °C. The ubiquitinated form of INF2 was detected by western blotting with an anti-HA antibody.

### Co-immunoprecipitation (co-IP) I

The HEK-293T cells were transfected with the indicated constructs. 36 h after transfection, the cells were treated with 20 μM MG132 for 8 h before harvesting. and then lysed in RIPA (Low) Lysis Buffer (Meilunbio, China, Cat No: MA0153). For immunoprecipitation, the cell lysates were incubated with anti-FLAG M2 agarose beads (Cat No. M8823) for 4 h at 4 °C. The bound beads were then washed four times with BC100 buffer (20 mM Tris-Cl, pH 7.9, 100 mM NaCl, 0.2 mM EDTA, 20% glycerol) containing 0.2% Triton X-100. The protein was eluted with FLAG peptide (Cat No. F4799) for 4 h at 4 °C. The results of the co-IP were detected by western blotting using the corresponding primary antibodies.

### Co-immunoprecipitation (co-IP) II

AN3 CA cells were lysed in RIPA (Low) lysis buffer (Meilunbio, China, Cat No: MA0153). For immunoprecipitation, the cell lysates were incubated with anti-FBXO7/IgG Protein A/G immunoprecipitated magnetic beads (Bimake, China, Cat No: B23201) for 4 h at 4 °C. The protein was eluted with SDS-PAGE loading buffer for 5 min at 100 °C. The results of the co-IP were detected by western blotting using the corresponding primary antibodies.

### Protein complex purification

An epitope-tagging strategy to isolate INF2-containing protein complexes from human cells. Briefly, HEK-293T cells were transfected with pCMV-FLAG-INF2 constructs. Tagged INF2 protein levels were detected by western blot analysis. For purification, cells were lysed in RIPA (Low) Lysis Buffer (Meilunbio, China, Cat No: MA0153) containing fresh protease inhibitor (Bimake, China, Cat No: B14001) on ice for 2 h. The homogenate was centrifuged for 30 min at 12,000 rpm at 4 °C. Cleared lysates were filtered through 0.45 μm spin filters (Millipore, USA, Cat No. SLHV033RB), and immunoprecipitated using anti-FLAG M2 agarose beads (Cat No. M8823). The bound polypeptides were eluted with the FLAG peptide (Cat No. F4799). The final elutes from the beads containing FLAG peptide were resolved by SDS-PAGE for Coomassie Blue staining.

### Flow cytometry

Flow cytometry was performed using Annexin V-FITC/PI apoptosis kit (MULTI SCIENCES, Cat No: AT101), according to the manufacturer’s instructions.

### Immunofluorescence and confocal microscopy

For immunofluorescence, the cells were plated on chamber slides and fixed with 4% paraformaldehyde at room temperature for 30 min. After washing with PBS, cells were permeabilized with 0.1% Triton X-100 in PBS for 15 min. The cells were then washed with PBS, blocked with 2% BSA in PBS for 1 h, and incubated with primary antibodies in PBS at 4 °C overnight. After washing with PBS, fluorescence-labeled secondary antibodies were applied and DAPI was counterstained for 1 h at room temperature. Cells were visualized and imaged using a confocal microscope (LEICA TCS SP8 STED).

For the analysis of mitochondrial length and morphology, cells were treated with Mito-Tracker (Thermo Fisher, USA, Cat No. M7512) at 37 °C in the dark for 30 min, and then performed as described above. The analytical methods for mitochondrial length and DRP1 puncta have been described previously [16, 18].

### Cell proliferation assay

Cell growth curves were generated using the CCK-8 method. Cell proliferation rates were determined using Cell Counting Kit-8 (CCK-8) (APExBIO, USA, Cat No. K1018) according to the manufacturer’s protocol. Cells were seeded in 96-well plates at a density of 2000 cells per well. On each of the 4 consecutive days of seeding, 10 µL of CCK-8 solution was added to each cell culture and incubated for 2 h. The resulting color was measured at 450 nm wavelength using a microplate absorbance reader (Bio-Rad, Hercules, CA, USA). Each measurement was performed in triplicates.

### Colony formation assay

A total of 2000 cells in 2 mL growth medium were plated in triplicate in the six-well plates, and the growth medium was changed every 3–4 days. After 14 days, the cells were rinsed twice with PBS, fixed with 10% formaldehyde, and stained with crystal violet (Solarbio, China, Cat No. G1063). The number of colonies was counted.

### Migration assays

Cell migration was determined using Transwell (Cat No. 3422; Costar, USA). The AN3 CA and HEC-1-A cell lines were cultured in a serum-free medium for 48 h. For the migration assay, 3 × 10^4^ cells were seeded in a serum-free medium in the upper chamber, and the lower chamber was filled with a growth medium containing 5% FBS. After 48 h, the non-migrating cells in the upper chambers were carefully removed with a cotton swab, and the migrated cells underside of the filter were stained and counted in nine different fields.

### Data acquisition

Data of the *FBXO7* RNA level and the FBXO7 protein level in ECa were respectively obtained from The Cancer Genome Atlas (TCGA) samples and Clinical Proteomic Tumor Analysis Consortium (CPTAC). Further analyses including differential expression analyses, clinical staging analyses, prognostic analyses and ROC analyses were performed by using software R (version 4.2.1).

### Immunohistochemistry

Human ECa tissues (*n* = 20) and corresponding normal endometrial tissues were obtained from Ningbo Clinical Diagnostic Pathology Center. Tissue microarrays (TMAs) were made using above 102 samples, including five normal tissues and 97 ECa tissues. Informed consent was obtained from all human participants, and the studies were approved by the Ethics Review Committee of Ningbo University. The protocols of immunohistochemistry and score have been described previously [34].

### Statistical analysis

Statistical calculations were performed using GraphPad Prism software. Data are presented as mean ± SD for experiments performed with at least three replicates. The differences between the two groups were analyzed using Student’s *t*-test, and multiple comparisons were performed using two-way analysis of variance (ANOVA). * represents *p* < 0.05; ** represents *p* < 0.01; *** represents *p* < 0.001.

## Supplementary information

Supplementary Information

## Data Availability

The datasets and computer code produced in this study are available in the following databases: The Cancer Genome Atlas (TCGA): https://portal.gdc.cancer.gov/. Clinical Proteomic Tumor Analysis Consortium (CPTAC): https://proteomics.cancer.gov/programs/cptac cBioportal for Cancer Genomics: http://www.cbioportal.org/. Protein Bank Data (PDB) https://www.rcsb.org/.
