# Supplementary material for: FBXO7, a tumor suppressor in endometrial carcinoma, suppresses INF2-associated mitochondrial division
Source: Cell Death Dis. 2023 Jun 21;14(6):368. doi: 10.1038/s41419-023-05891-0 (PMC10284917; doi:10.1038/s41419-023-05891-0)
Supplement: Supplementary file 1 — Supplementary Information [file 41419_2023_5891_MOESM1_ESM.docx]

Supplementary Information for

**FBXO7, a tumor suppressor in endometrial carcinoma,**

**suppresses INF2-** **associated mitochondrial division**

Hui Zhang^1,2^, Yiting Zhao^1,2^, Jie Wang^2^, Jinyun Li^2^, Jingyi Xia^1^, Yan Lin^1,2^, Yeling Zhong^1,2^, Xinyi Cao^1^, Jiabei Jin^1^, Xinming Li^1^, Weili Yang^3^, Meng Ye^2^, Xiaofeng Jin^1,2, #^

**This PDF file includes:**

Supplementary Fig. 1: INF2 is a specific substrate of FBXO7

Supplementary Fig. 2: ECa-associated FBXO7 mutants

Supplementary Fig. 3: FBXO7-ΔUBL is deficient in the ubiquitination and degradation of INF2

Supplementary Fig. 4: Identify the key domains of FBXO7-INF2 interaction

Supplementary Fig. 5: FBXO7 suppresses HEC-1-A cells proliferation and migration partly in an INF2-DRP1 axis-dependent manner

Supplementary Fig. 6: FBXO7 is involved in mitochondrial division and fusion

Supplementary Fig. 7: FBXO7 is the promoter of mitophagy

Supplementary Fig. 8: FBXO7-INF2-DRP1 axis involves in the apoptosis of ECa cells

Supplementary Table 1: The primers sequence

Supplementary Table 2: Sequences of sgRNA and siRNA targeted sequences

Supplementary Table 3: Antibody and Chemicals

**
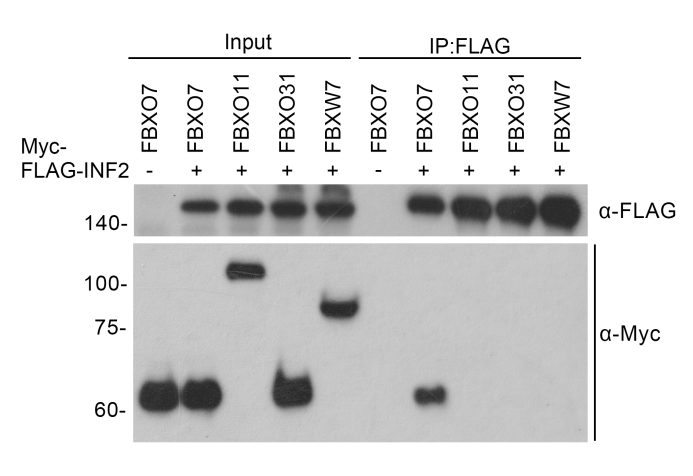
**

**Supplementary Fig. 1: INF2 is a specific substrate of FBXO7**

Western blotting of WCLs and co-IP samples of anti-FLAG antibody obtained from HEK-293T cells transfected with Myc-FBXO7/FBXO11/FBXO31/FBXW7 plasmids and/or not FLAG-INF2-WT plasmid, and treated with 20 μM MG132 for 8 h before harvesting. Experiments were repeated three times.


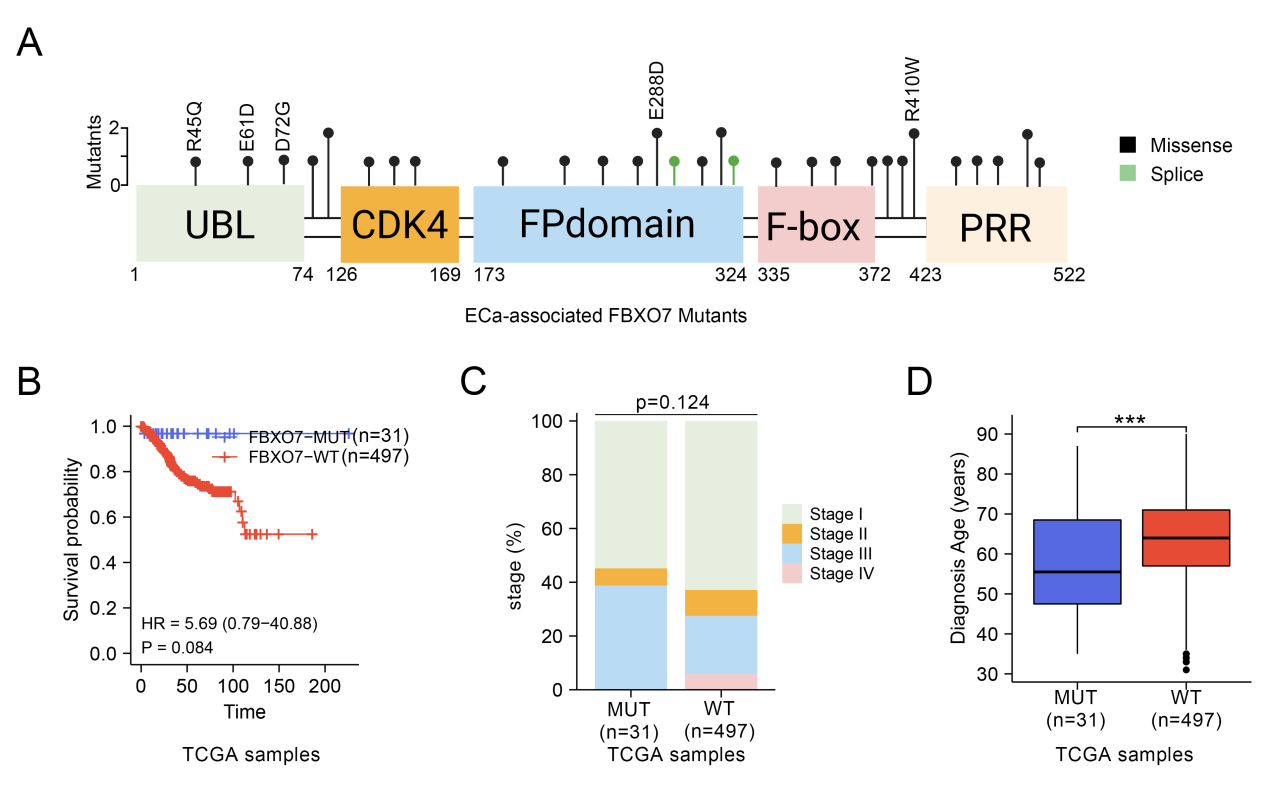


**Supplementary Fig. 2: ECa-associated FBXO7 mutants**

1. Diagram showing ECa-associated FBXO7 mutants (n = 31) from cBioportal database.
2. Prognosis analysis of ECa patients with FBXO7-WT (n = 497) and FBXO7-MUT (n = 31) from TCGA database using R software.
3. Clinical staging analysis of ECa patients with FBXO7-WT (n = 497) and FBXO7-MUT (n = 31) from TCGA database using R software.
4. Diagnosis age analysis of ECa patients with FBXO7-WT (n = 497) and FBXO7-MUT (n = 31) from TCGA database. ***p < 0.001 vs. the Normal group.


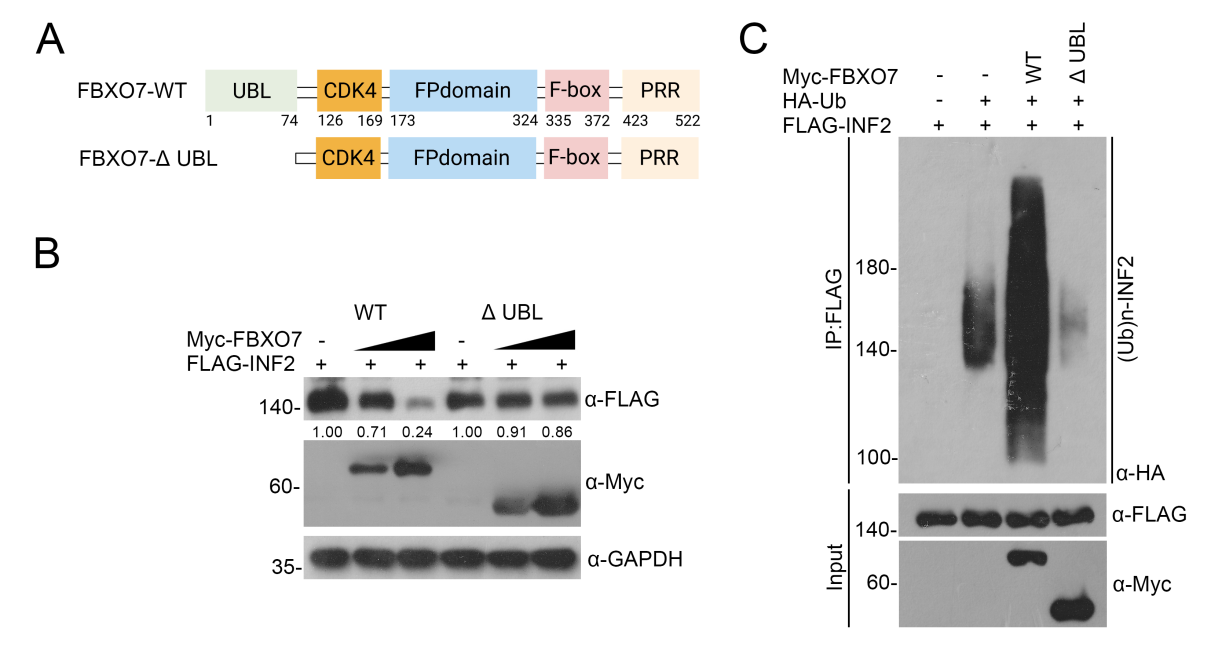


**Supplementary Fig. 3: FBXO7-ΔUBL is deficient in the ubiquitination and degradation of INF2**

1. Diagram showing wild-type FBXO7 protein and FBXO7 protein deletion of UBL domain (FBXO7-ΔUBL).
2. Western blotting of WCLs of HEK-293T cells transfected with FLAG-INF2 plasmid and the increasing Myc-FBXO7-WT/ΔUBL plasmids. All quantitation were normalized to the protein level of endogenous control GAPDH.
3. Western blotting of the products of *in vivo* ubiquitination assays performed using WCLs and co-IP samples of anti-FLAG antibody from HEK-293T cells transfected with the FLAG-INF2 plasmid, and/ or not transfected with Myc-FBXO7-WT/ΔUBL and HA-Ub plasmids,and treated with 20 μM MG132 for 8 h before harvesting.

Experiments in **(B, C)** were repeated three times.


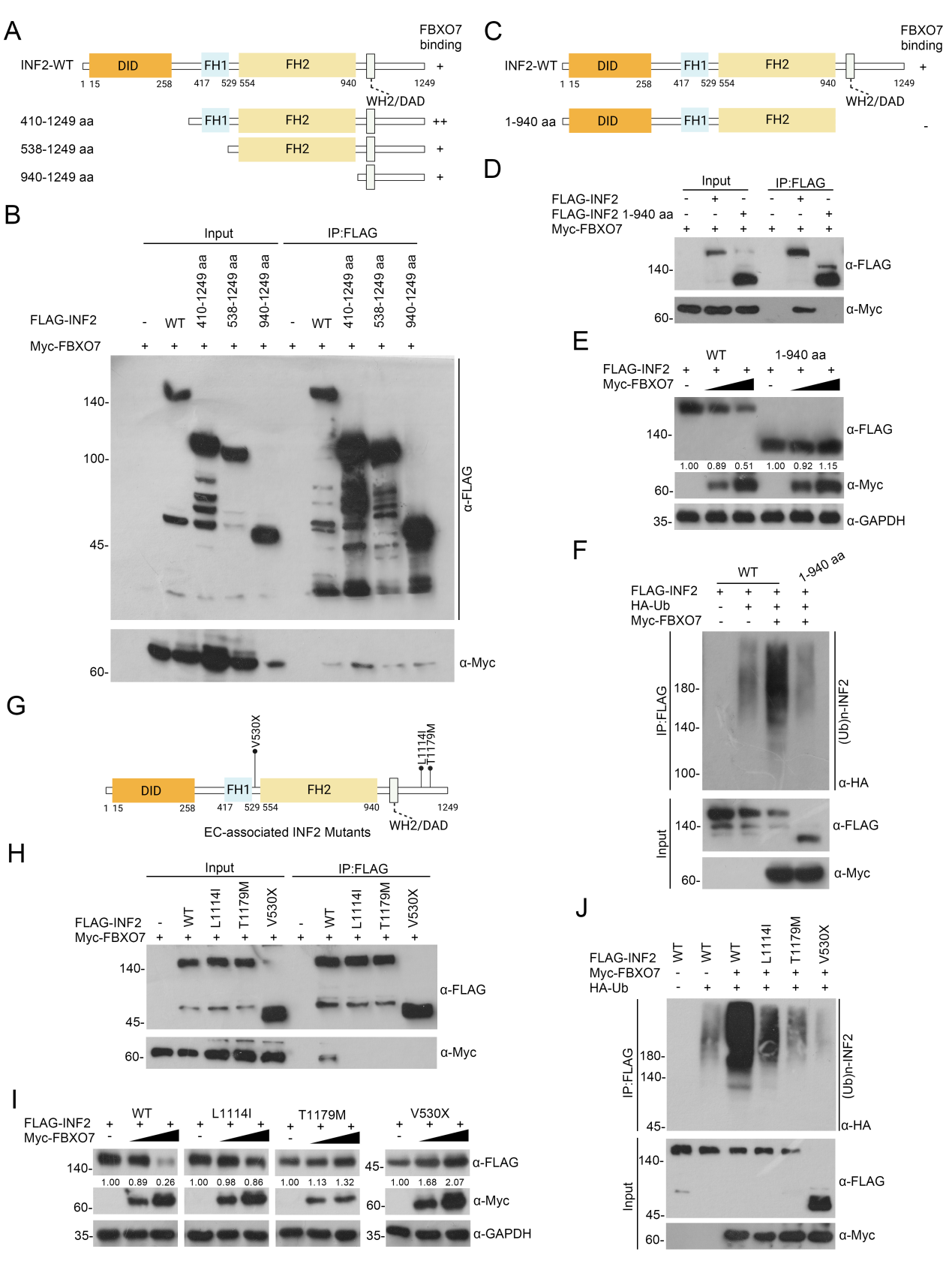


**Supplementary Fig. 4: Identify the key domains of FBXO7-INF2 interaction**

1. Diagram showing INF2-WT and INF2 structural domain truncation mutants.
2. Western blotting of WCLs and co-IP samples of anti-FLAG antibody obtained from HEK-293T cells transfected with Myc-FBXO7 plasmid and/or not FLAG-INF2-WT/410-1249 aa/538-1249 aa/940-1249 aa plasmids, and treated with 20 μM MG132 for 8 h before harvesting.
3. Diagram showing INF2-WT and INF2 1-940 aa.
4. Western blotting of WCLs and co-IP samples of anti-FLAG antibody obtained from HEK-293T cells transfected with Myc-FBXO7 plasmid and/or not FLAG-INF2-WT/1-940 aa plasmids, and treated with 20 μM MG132 for 8 h before harvesting.
5. Western blotting of WCLs of HEK-293T cells transfected with FLAG-INF2-WT/1-940 aa plasmids and gradient increasing Myc-FBXO7-WT plasmid. All quantitation were normalized to the protein level of endogenous control GAPDH.
6. Western blotting of the products of *in vivo* ubiquitination assays performed using WCLs and co-IP samples of anti-FLAG antibody from HEK-293T cells transfected with the FLAG-INF2-WT/1-940 aa plasmids, and/or not transfected with Myc-FBXO7-WT and HA-Ub plasmids, and treated with 20 μM MG132 for 8 h before harvesting.
7. Diagram showing ECa-associated INF2 mutants from cBioportal database.
8. Western blotting of WCLs and co-IP samples of anti-FLAG antibody obtained from HEK-293T cells transfected with Myc-FBXO7 plasmid and/or not FLAG-INF2-WT/L1114I/T1179M/V530X plasmids, and treated with 20 μM MG132 for 8 h before harvesting.
9. Western blotting of WCLs of HEK-293T cells transfected with FLAG-INF2-WT/L1114I/T1179M/V530X plasmids and the increasing Myc-FBXO7-WT plasmid. All quantitation were normalized to the protein level of endogenous control GAPDH.
10. Western blotting of the products of *in vivo* ubiquitination assays performed using WCLs and co-IP samples of anti-FLAG antibody from HEK-293T cells transfected with the FLAG-INF2-WT/L1114I/T1179M/V530X plasmids, and/or not transfected with Myc-FBXO7-WT and HA-Ub plasmids, and treated with 20 μM MG132 for 8 h before harvesting.

Experiments in **(B, D, E, F, H, I, J)** were repeated three times.


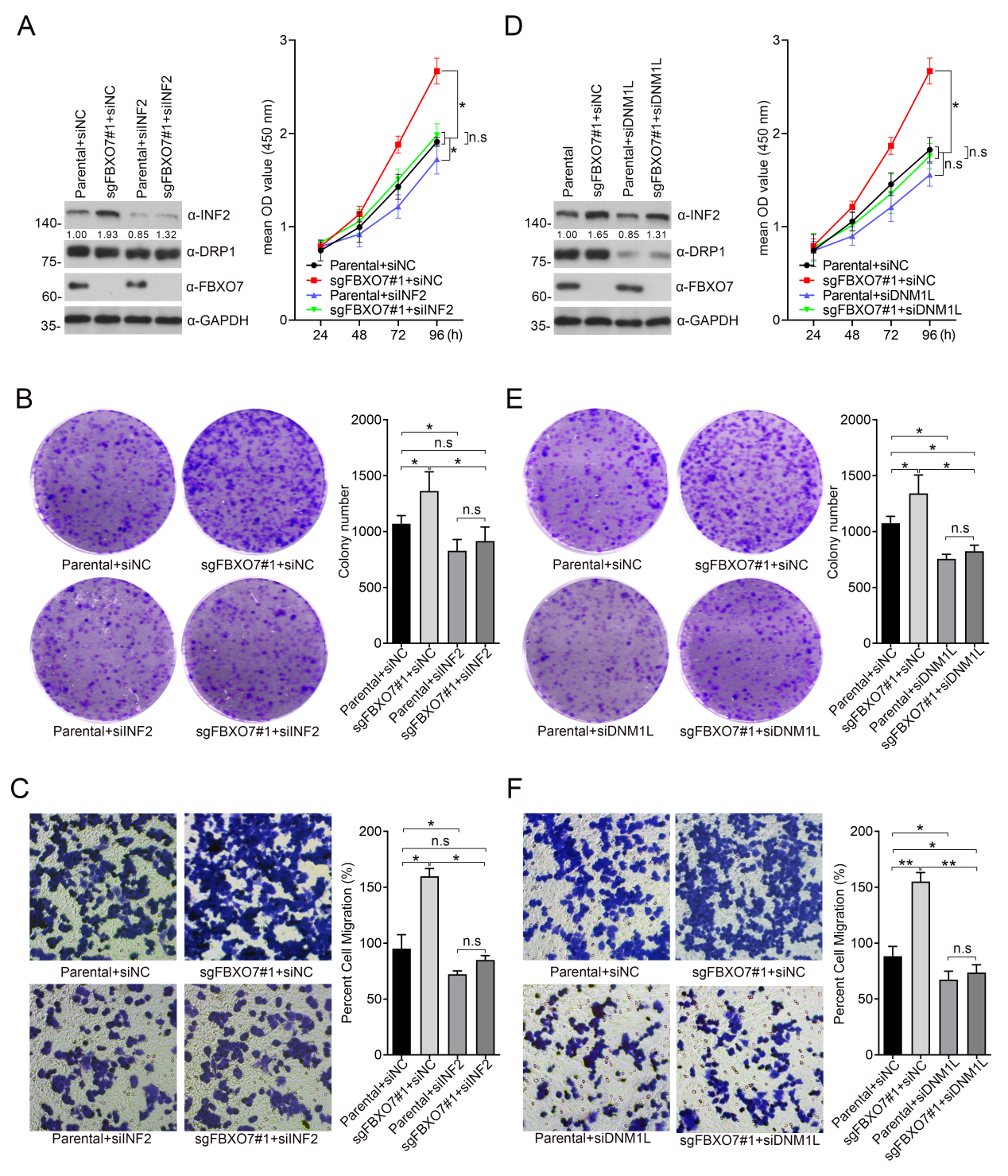


**Supplementary Fig. 5: FBXO7 suppresses HEC-1-A cells proliferation and migration partly in an INF2-DRP1 axis-dependent manner**

1. Western blotting of WCLs of HEC-1-A cells with FBXO7 knockout and infected with siNC RNA, or siINF2 RNA, or parental (Left). All quantitation were normalized to the protein level of endogenous parental GAPDH. Cell proliferation assay of HEC-1-A cells with FBXO7 knockout and infected with siNC RNA, or siINF2 RNA, or parental (Right). Data are shown as means ± SD (n = 5). *p < 0.05.
2. Cell colony formation assay of HEC-1-A cells with FBXO7 knockout and infected with siNC RNA, or siINF2 RNA, or parental (Left). Statistics of cell colony formation assay (Right). Data are shown as means ± SD (n = 3). *p < 0.05.
3. Cell migration assay of HEC-1-A cells with FBXO7 knockout and infected with siINF2 RNA, or siINF2 RNA, or parental (Left). Statistics of cell migration assay (Right). Data are shown as means ± SD (n = 3). *p< 0.05.
4. Western blotting of WCLs of HEC-1-A cells with FBXO7 knockout and infected with siNC RNA, or siDNM1L RNA, or parental (Left). All quantitation were normalized to the protein level of endogenous parental GAPDH. Cell proliferation assay of HEC-1-A cells with FBXO7 knockout and infected with siNC RNA, or siDNM1L RNA, or parental (Right). Data are shown as means ± SD (n = 5). *p < 0.05.
5. Cell colony formation assay of HEC-1-A cells with FBXO7 knockout and infected with siNC RNA, or siDNM1L RNA, or parental (Left). Statistics of cell colony formation assay (Right). Data are shown as means ± SD (n = 3). *p < 0.05.
6. Cell migration assay of HEC-1-A cells with FBXO7 knockout and infected with siDNM1L RNA, or siDNM1L RNA, or parental (Left). Statistics of cell migration assay (Right). Data are shown as means ± SD (n = 3). *p< 0.05, **p< 0.01.

Experiments in **(A, D)** were repeated three times.


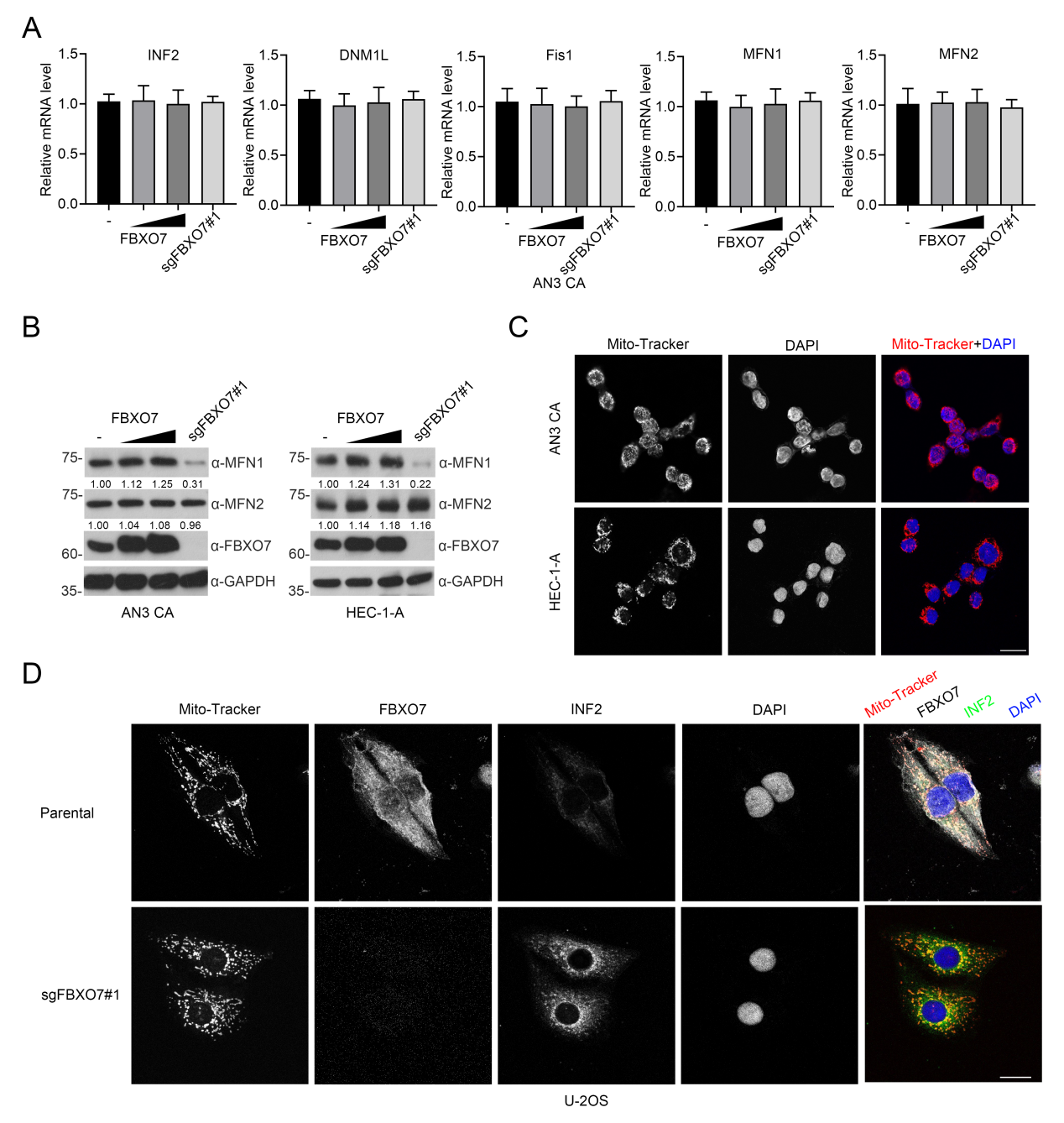


**Supplementary Fig. 6: FBXO7 is involved in mitochondrial division and fusion**

1. The mRNA expression level of *INF2*, *DNM1L*, *Fis1*, *MFN1* and *MFN2* in AN3 CA cells with *FBXO7* knockout and FBXO7 overexpression. All quantitation were normalized to the mRNA level of endogenous *GAPDH*.
2. Western blotting of WCLs of AN3 CA and HEC-1-A cells with *FBXO7* knockout and FBXO7 overexpression to detect the protein level of MFN1 and MFN2. All quantitation were normalized to the protein level of endogenous parental GAPDH. Experiments were repeated three times.
3. Images of AN3 CA and HEC-1-A cells. stained with Mito-Tracker Red and DAPI. Scale bar, 20 μm.
4. Images of U-2OS cells with *FBXO7* knockout and parental. stained with Mito-Tracker Red, INF2, FBXO7 and DAPI. Scale bar, 20 μm.


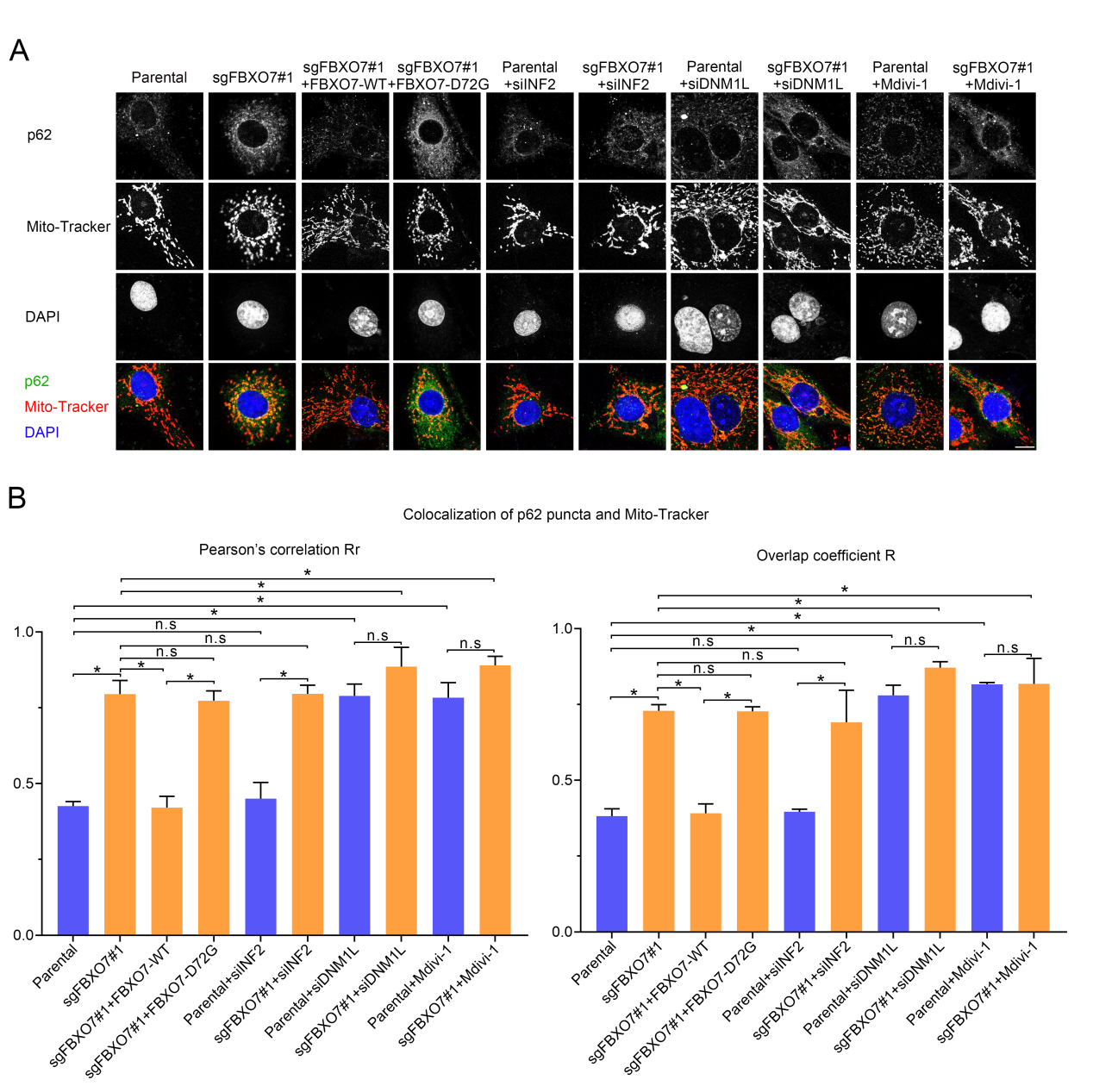


**Supplementary Fig. 7: FBXO7 is the promoter of mitophagy**

1. Representative images of U-2OS cells with *FBXO7* knockout or parental transfected with pCDH-CD513B-FBXO7-WT/D72G plasmids, si*INF2* RNA, si*DNM1L* RNA, or treated with Mdivi-1 (20 μM). stained with p62, Mito-Tracker Red and DAPI. Scale bar, 20 μm.
2. Quantitative analysis of colocalization among p62 puncta and Mito-Tracker through Image Pro Plus 6.0 sofrware. Data are shown as means ± SD (n = 5). *p < 0.05.


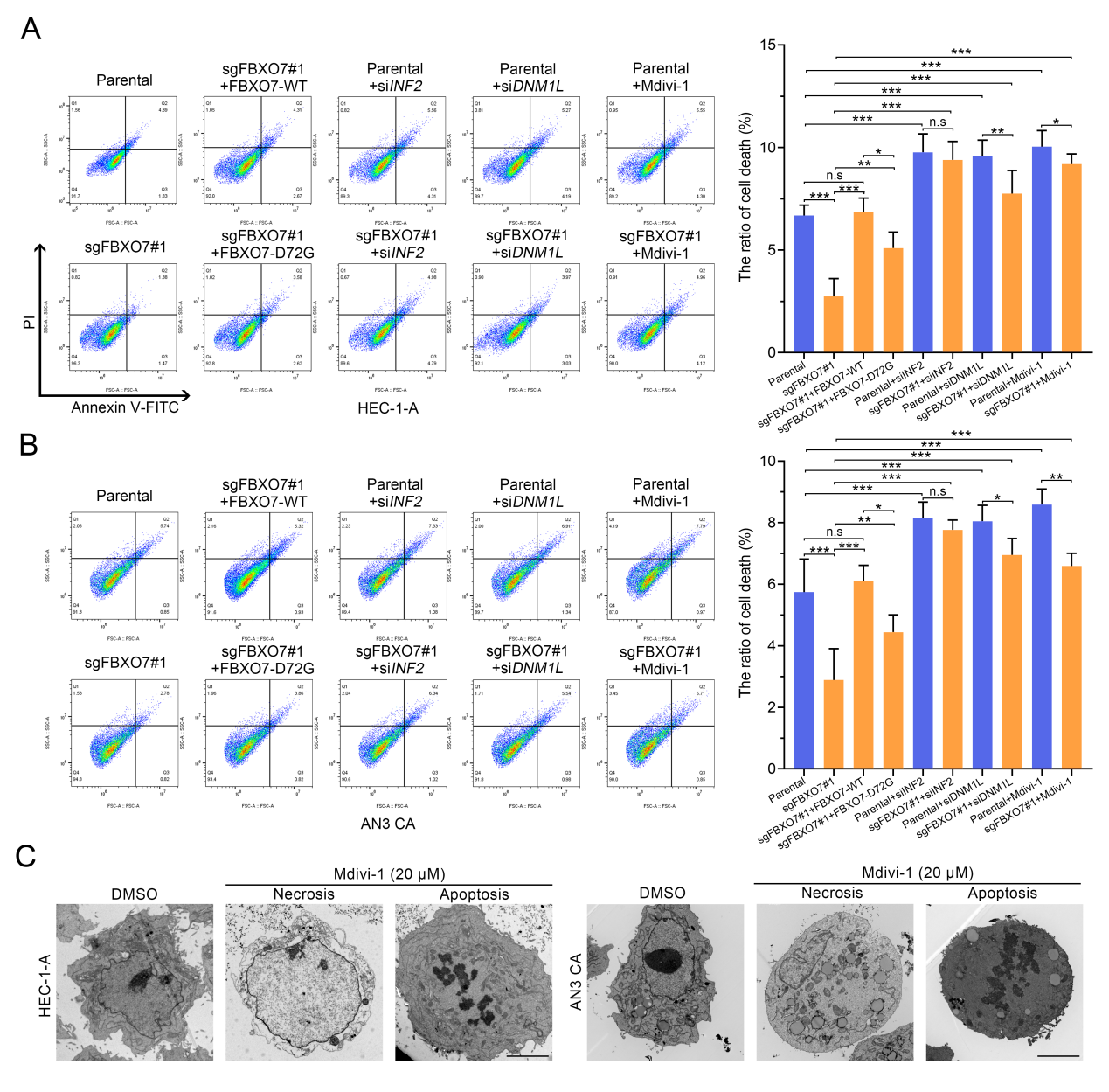


**Supplementary Fig. 8: FBXO7-INF2-DRP1 axis involves in the apoptosis of ECa cells**

1. The apoptosis level of HEC-1-A cells with *FBXO7* knockout or parental transfected with pCDH-CD513B-FBXO7-WT plasmid, pCDH-CD513B-FBXO7-D72G plasmid, si*INF2* RNA, si*DNM1L* RNA, or treated with Mdivi-1 (20 μM) detected by flow cytometry. Data are shown as means ± SD (n = 6). *p < 0.05, **p < 0.01, ***p < 0.005.
2. The apoptosis level of AN3 CA cells with *FBXO7* knockout or parental transfected with pCDH-CD513B-FBXO7-WT plasmid, pCDH-CD513B-FBXO7-D72G plasmid, si*INF2* RNA, si*DNM1L* RNA, or treated with Mdivi-1 (20 μM) detected by flow cytometry. Data are shown as means ± SD (n = 6). *p < 0.05, **p < 0.01, ***p < 0.005.
3. Transmission electron microscopy analysis of photomicrographs of HEC-1-A and AN3 CA cells treated with Mdivi-1 (20 μM) or DMSO. Scale bar, 5 μm.

**Supplementary Table 1: The primers sequence**

| Construction Primers | |
| --- | --- |
| Gene | Sequence 5’-3’ |
| FBXO7 | F: AAAGAATTCGCATGAGGCTGCGGGTGCGGCTTCTGAA  R: AAAGCGGCCGCTCACATGAATGACAGCCGGCCATCAGTTG |
| INF2 | F: AAAGAATTCGCATGTCGGTGAAGGAGGGCGCA  R: AAACTCGAGTCACTTGGCCTTGGGCCTGGGCCTGAGGC |
| FBXO7  Δ UBL | F: AAAGAATTCGCTGTTTGATTCTTCAAGATGACATTCCAGC |
| FBXO7  1-74 AA | R: AAAGCGGCCGCTCATATCAAGTCCCCAGAAACAA |
| FBXO7  1-169 AA | R: CTGCCGTTCGACGATGCGGCCGCTCACATGGGTTCTGAGGGATAGA |
| FBXO7  169-381 AA | F: AAAGAATTCGCCTCTGTAGTGAATCGGTGGAAGGCAA |
| FBXO7  1-381 AA | R: CTGCCGTTCGACGATGCGGCCGCTCGAAAATCACGCAGATATAAAAAC |
| FBXO7  R45Q | F: CAGTTCTAATACCCAATTTACAATTACAT  R: TAGTTCAATGTAATTGTAAATTGGGTATTAG |
| FBXO7  E61D | F: CCCCTCACTGGAGATGAAGACACCTTGGCTTC  R: CCCATATGAAGCCAAGGTGTCTTCATCTCCA |
| FBXO7  D72G | F: TATGGGATTGTTTCTGGGGGCTTGATATGTTTG  R: AAGAATCAAACATATCAAGCCCCCAGAAACAATC |
| INF2  1-940 AA | R: AAAGCGGCCGCTTACCTCCTCTCTGCCTTCGCCGCCTG |
| INF2  V530X | R: AAAGCGGCCGCTTAGGGGGGGCTGCAGGTGCAGG |
| INF2  L1114I | F: GAGATGCTCAGGCCCTGAAGCCCATCAAGTTCTCCAGCA  R: GCAGGGGGCTGGTTGCTGGAGAACTTGATGGGCTTCAGGGCCTGAG |
| INF2  T1179M | F: ATGAGGACGAGGACGAGGAGGACATGGCCCCAGAGTCCGCACTG  R: TTGTCCAGGGATGTGTCCAGTGCGGACTCTGGGGCCATGTCCTCCTCGTCCTCG |

**Supplementary Table 2: Sequences of sgRNA and siRNA targeted sequences**

| Gene | Sequence 5’-3’ |
| --- | --- |
| sgFBXO7 | F: GTCTGGTGTTTGGAATGACG |
| siINF2 | F: ACAAAGAAACTGTGTGTGTGA |
| siDNM1L | F: CCCTAGCTGTAATCACTAA |

**Supplementary Table 3: Antibody and Chemicals**

| No. | Name | Species | Cat No. | Source |
| --- | --- | --- | --- | --- |
| 1 | Anti-FBXO7 | Rabbit | 10696-1-AP | Proteintech |
| 2 | Anti-INF2 | Rabbit | A10038 | Abclonal |
| 3 | Anti-INF2 | Mouse | 66910-1-Ig | Proteintech |
| 4 | Anti-DRP1 | Rabbit | ab184247 | abcam |
| 5 | Anti-GAPDH | Rabbit | AC001 | Abclonal |
| 6 | Anti-Myc | Mouse | M192-7 | MBL |
| 7 | Anti-FLAG | Mouse | M185-7 | MBL |
| 8 | Anti-HA | Mouse | M180-7 | MBL |
| 9 | Anti-FLAG M2 agarose beads | Mouse | M8823 | Sigma |
| 10 | Anti-Parkin | Rabbit | A11172 | Abclonal |
| 11 | Anti-MFN1 | Rabbit | A9880 | Abclonal |
| 12 | Anti-MFN2 | Rabbit | A12771 | Abclonal |
| 13 | Anti-p-DRP1 Ser616 | Rabbit | 3455 | CST |
| 14 | Anti-p-DRP1 Ser637 | Rabbit | 4867 | CST |
| 15 | FLAG peptide |  | F4799 | Sigma |
| 16 | MG132 |  | A2585 | APExBIO |
| 17 | Mdivi-1 |  | A4427 | APExBIO |
| 18 | CHX |  | HY-B0713 | MCE |
| 19 | Mito-Tracker |  | M7512 | Thermo Fisher |
| 20 | Bortezomib |  | S1013 | Selleckchem |
| 21 | Chloroquine |  | HY-17589A | MCE |
